# Sentiment trajectory modeling in mental health discussions via transformer-based analysis

**DOI:** 10.3389/frai.2026.1810552

**Published:** 2026-06-26

**Authors:** Shaina U, Tamilarasi Kathirvel Murugan, Akshaya Poorna R, Logeswari Govindaraj, Joel Prince

**Affiliations:** School of Computer Science and Engineering, Vellore Institute of Technology, Chennai, India

**Keywords:** early detection, mental health, natural language processing, Reddit, sentiment analysis, social media, temporal sentiment progression analysis, transformer models

## Abstract

**Introduction:**

Social media platforms produce a constant stream of user-generated text, which holds promise as a source of useful information regarding population-level and individual mental health states. However, existing methods for sentiment analysis on social media texts process these texts as individual, static instances, failing to consider the underlying dynamics of emotional states, which are naturally evolving in nature.

**Methods:**

In this paper, we introduce a novel framework for Temporal Sentiment Progression Analysis, which uses domain-specific transformer architectures to reconstruct the entire emotional evolution of a user over a given period of time. This framework captures important aspects of emotional evolution, including variability, volatility, and key emotional inflection points in a user’s emotional progression. Our approach performs per-comment sentiment and thematic classification using transformer models, followed by post hoc statistical analysis to examine temporal patterns in user discussions. The framework was evaluated on a dataset of approximately 4,000 Reddit comments collected from eight mental health-related subreddits, with additional synthetic samples used only during training to address class imbalance. Performance evaluation was conducted exclusively on authentic Reddit comments and further validated using external Reddit-based mental health datasets and comparative baseline experiments.

**Results:**

Our experimental results on a large-scale social media dataset reveal different sentiment progression archetypes, which are strongly correlated with self-reported mental health concerns. Our proposed framework achieves a high classification accuracy of 92%.

**Discussion:**

Our approach shifts from static sentiment analysis to dynamic sentiment progression analysis, which can help in understanding the evolution of emotional distress in a more nuanced manner and can be useful in the development of context-aware interventions for mitigating mental health concerns.

## 1 Introduction

### 1.1 Motivation

Digital Life as a Window into Mental Health. Mental health disorders are a growing and high health problem on a global scale. Major Depressive Disorder (MDD), for instance, impacts about 5% of the global adult population. Even more alarming, nearly a third of individuals will navigate a clinically significant depressive episode at some stage, according to recent evidence (Tadesse et al., 2019). The results reach far beyond the individual; depression is now the most frequent cause of global disability that accounts, annually, for up to an economic injury of nearly a trillion US dollars (Kabir et al., 2023). Yet there is still a divide in the body that separates those struggling with it from those getting treatment. Barriers to healthcare access, and the lasting burden of social taboo and the secret of psychological grief have resulted in many unrecorded and neglected cases (Tadesse et al., 2019; Kabir et al., 2023). This persistent void has led to the pursuit of complementary, novel avenues to support, where computational approaches have emerged as the promising path forward (Kumar et al., 2023).

As a result of this public health crisis, social media platforms have burgeoned over the last 20 years. Websites such as Reddit, X (formerly Twitter), and Weibo are de facto public squares where billions of users express emotional experiences, share personal stories, and network around similarities and disagreements (Guo et al., 2023; Eberhardt et al., 2025). Unlike fast-tracked-fire conversations, these forums often allow for a mixture of anonymity and intimate, long-form, personal narrative (Morini et al., 2025), which can be an advantage in delicate discussions about mental health. In consequence, these online channels have become extremely useful repositories of longitudinal, organic data of human activity. It allows scientists to gain an unprecedented and unique perspective on mental health as it manifests in a real-world context, devoid of the pressure of the clinical context (Tadesse et al., 2019; Abdullah and Negied, 2024). It is this clash between an urgent public health exigency and a ubiquitous source of data that is the central motivator for the identification of advanced computational modalities. It is not meant to take the place of human care, but to make use of this digital footprint to make better public health insight possible, early risk indication and intervention in risks (Kim et al., 2020; Tadesse et al., 2019).

### 1.2 Background: computational language analysis for mental health

The course of computational linguistic analysis of mental health detection has followed the trajectory of the natural language processing (NLP) revolution (Dubey et al., 2025). At first, existing algorithms in the field were largely based on traditional machine learning, such as SVM, Logistic Regression and Random Forests, with their great need for careful manual labor (Tadesse et al., 2019; Yates et al., 2017). At this point, researchers increasingly relied on existing psychological dictionaries, particularly the Linguistic Inquiry and Word Count (LIWC), to extract elements associated with psychological constructs, for example, emotional tones or social themes (Kabir et al., 2023). While these models provided a clear rationale for their decisions, their ability to view the world through a narrow filter was constricted by the dictionaries they were using. They could only see parts of what they had been trained to scour, and risked seeing too many of the same patterns—and then missing subtle, new ones—of distress that would seem to be missing in the online world.

Deep learning brought a paradigm shift. Architectures such as Convolutional Neural Networks (CNNs) and LSTMs started learning feature representations from raw text directly with an increase in pre-trained word representations, such as Word2Vec and GloVe (Chiong et al., 2021; Wang et al., 2020; Scherbakov et al., 2025). LSTMs have especially made progress in dealing with the narrative-like flow of language. However, their sequential design was cumbersome, and more critically, they struggled to connect distant yet related thoughts across a user’s long posting history—a significant disadvantage for examining the subtleties of time or a person’s mental state (Lin et al., 2020; Murarka et al., 2021).

True breakthroughs came with a transformer architecture, epitomized by BERT and subsequent models (Kurniadi et al., 2024). Using a “self-attention” mechanism, these models process words in relation to all other words in a text at once. By reading parallel text, these models show a potential for understanding complex and long-distance contextual relationships that previous models overlooked, resulting in a much more sophisticated interpretation of how psychological distress is conveyed in language (Trotzek et al., 2018; Lin et al., 2020). The state-of-the-art data collection performance of these models in mental health detection tasks is impressive (Yates et al., 2017; Denecke and Deng, 2015). Recent studies have remained increasingly convincing: for instance, BERT-based models have shown superior accuracy, for example, up to 94.89%, in identifying indicators of depression in Reddit posts (Rozado et al., 2022). The breadth of their application has also expanded to include multiple disorders, such as ADHD, Bipolar Disorder, and PTSD, as well, with remarkable reliability (Denecke and Deng, 2015).

### 1.3 Research gap: from static detection to dynamic sentiment trajectories

Although modern transformer models are more sophisticated, most existing research focuses only on static classification (Tadesse et al., 2019; Chiong et al., 2021). The dominant goal of many studies is to ascertain whether, based on a single post or a cumulative archive of a user’s historical posts, that user can be classified as part of a group with a mental health condition (Ren et al., 2025; Nagata et al., 2025; Borrego-Ruiz and Borrego, 2025). Useful for early screening, this technique essentially boils down a person’s complex psychological journey to some sort of categorical tag instead of creating a dynamic picture.

A clearer and fresher direction is a more recent perspective on change over time toward early detection (Kabir et al., 2023; Cong et al., 2024). By analyzing the naturally chronological nature of social media, researchers hunt for shifts in language that could signal a user’s later disclosure of a diagnosis. For example, previous studies have shown that the 12–16 weeks leading up to a depression detection are particularly informative, where negative emotional terminology spiked, often within a clear 4- to 8-week window (Cong et al., 2024). It reinforces the truth that the road toward a crisis frequently bears a linguistic footprint. A similar technical feasibility for long-term tracking is found in other contexts, such as sentiment analysis in millions of news headlines over 20 years with transformer models (Merchant et al., 2019). To promote that line of inquiry, high-quality, clinically validated longitudinal data is particularly crucial to date. The 3S-YP study is an important milestone in bridging social media data to electronic health records (Cao et al., 2024).

However, even these sophisticated studies of timestamped data tend to break a user’s history down into chunks and analyze each one separately. This implicitly considers each time window as a data point of its own, and does not directly model the ongoing trajectory of a person’s emotional state. The lack of a formal framework to model the sentiment trajectory is the critical research gap, for instance. The idea is gaining traction; researchers have worked up models that, based on the sentiment curve of individual users on Weibo, have started identifying negative trends (Gan et al., 2025). The clinical application of this strategy has also been validated; it was observed that the clinical benefits were improved in patients with a general upward trend in sentiment in psychotherapy transcripts (Arif et al., 2024). Thereby, the central question needs to change from: “Are you going to catch depression early?” to interrogating, “What does the journey look like?” What are the patterns—the shapes, speed and turbulence—of someone navigating their emotional life as they reach a crisis, come to terms with/solve it and perhaps even recover from it? Addressing this limitation requires a trajectory-oriented analytical approach that moves beyond static snapshots toward examining temporal patterns of emotional change over time.

Some recent research works have also addressed the issue of exploring the temporal dynamics in the online mental health discussions through the use of predictive temporal windows and sequential modeling methods for the detection of changing patterns of psychological risk signals. For example, the use of temporal predictive windows for the detection of mental health risk in social media discussions has also been addressed in previous research works (Owen et al., 2023). However, in most of these research works, the use of time as a predictive feature has been considered, and in this research, a trajectory-oriented framework for the analysis of the evolution of mental health discussions is proposed. The framework also incorporates transformer-based sentiment analysis using RoBERTa in conjunction with domain-specific thematic mental health content classification using MentalBERT, thereby facilitating the detection of both emotional tone and disorder-related thematic cues. Beyond classification, the proposed system also incorporates statistical analysis for detecting the progression of emotions using measures such as betterment-deterioration transition scores, sentiment volatility measures, and non-parametric trend detection using the Mann-Kendall test, thereby facilitating the detection of emotional stability, episodic changes, and deterioration patterns in user discussion histories.

### 1.4 Key contributions and proposed framework

The goal of this article is to close the gap in the existing literature set forth above by presenting a unique framework for modeling and interpreting the sentiment paths in online mental health discourse. Our work contributes the following:

- *A Two-Stage Framework for Detection and Analysis*: We have developed a novel dual-transformer architecture that will initially search for consistent psychological distress, such as persistently common themes of sadness or anxiety, in a user’s post history using RoBERTa, a strong general-purpose language model. This permits rapid and accurate pre-alerting to potential issues, in informal language patterns present even in social media, which is inconsistent and not a systematic method (Kurniadi et al., 2024).
- *Diagnosis and Progression Tracking with MentalBERT:* To go from detection to identification, we next utilize MentalBERT as a part of our framework. This model is a transformer specially built for mental health language. Because of its sensitivity to clinical subtleties, we can construct an accurate and detailed map of a user’s mental health trajectory over time. This enables the analysis of changes in emotional expression and thematic discussion patterns over time, providing interpretable indicators of sentiment progression and temporal behavioral trends. Rather than implementing a fully end-to-end temporal deep learning framework, the proposed approach combines transformer-based linguistic analysis with interpretable *post-hoc* temporal statistical modeling to examine progression patterns in user discussions over time.

The proposed work integrates transformer-based sentiment and thematic classification with *post-hoc* temporal statistical analysis for examining progression patterns in mental health discussions. Unlike prior approaches that focus on static classification, the framework introduces interpretable progression metrics, including trend slope, sentiment volatility, and transition-based measures, to characterize temporal patterns in user-generated text. The proposed approach emphasizes analytical interpretability and temporal pattern identification rather than end-to-end trajectory modeling.

### 1.5 Organization of the paper

The remainder of this study is organized as follows: Section II describes the dataset construction, pre-processing steps, and ethical considerations. Section III details the proposed temporal sentiment progression analysis framework, including the dual-transformer architecture and trajectory extraction methodology. Section IV presents the experimental setup, evaluation metrics, and comparative results. Section V discusses the identified sentiment trajectory archetypes and their clinical implications. Finally, Section VI concludes the study and outlines directions for future research.

## 2 Literature review

### 2.1 Feature engineering foundations: the lexicon-based paradigm

Initial tries to study mental health with text were based on traditional machine learning techniques. Yet these methods met an existential challenge: language is raw, unstructured language. In order for a model such as SVM, Logistic Regression, or Random Forest (RFR) to predict whether someone belongs to a particular user group, researchers have first had to transform these freely flowing texts into organized numbers. This laborious process for building features became the core task of the discipline. Therefore, scholars heavily relied on the established psycholinguistic dictionaries; the LIWC dictionary emerged as a more prominent reference (Kabir et al., 2023). LIWC offers a method of quantifying language by its pre-determined psychological characteristics (such as by counting words of social relationships, emotions and cognitive processes; Kabir et al., 2023). These features of a language were typically supplemented with simple statistical text representations (e.g., n-grams), and data of users, being available for model input (Kim et al., 2020; Tadesse et al., 2019).

This is a major advantage as it provides clear meaning for us. The features were based on long-standing psychological theory, which enabled one to easily connect the model’s decision with a concrete linguistic pattern. However, such a strength was also its greatest weakness. All these approaches were limited to using pre-determined lexicons; they restricted themselves to an analytical box, meaning they only recognized patterns they were explicitly programmed for. This hindered them from finding organic and nuanced linguistic signs developing in the online mental health conversations. This limitation is further illustrated by the subsequent and persistent superiority of deep learning models that show transformers extract essential diagnostic signals that are beyond the bounds of traditional lexicons. So the transition to deep learning was not just an update of underlying technology; it was a leap that expanded the space of research.

### 2.2 The transition to data-driven feature discovery

The development of deep learning represented a turning point, where the area was taken from a manual curation process to automatic discovery. In contrast to the architectures that preceded them, CNNs and Recurrent Neural Networks (RNNs) avoided feature engineering in learning to recognize intricate, meaningful patterns as patterns from raw text (Chiong et al., 2021; Wang et al., 2020; Scherbakov et al., 2025). And each architecture had a particular edge: CNNs were powerful at picking out local, telling phrases, RNNs (especially Long Short-Term Memory (LSTM) networks) were built to take the stepwise stream of language, such as reading a sentence one word at a time.

One of the defining features of this epoch was the use of pre-trained word embeddings such as Word2Vec and GloVe (Chiong et al., 2021). By giving deep, semantically meaningful vector representations from the beginning, these embeddings allowed models to understand word meaning in the context of the full semantics of words. However, in the face of substantial gains in the predictive performance, the development of this model generation had important limitations. LSTM sequence features caused computational overhead and were not parallelizable by their sequential design. As a consequence, they were more vulnerable to the vanishing gradient problem, which limited their ability to understand long-range dependencies present in a text—crucial for understanding a user’s full expression of intent (Lin et al., 2020; Murarka et al., 2021). This is a huge disadvantage for mental health analysis, where the whole of a person’s profile needs to be understood from hundreds of posts. In this way, LSTMs imposed a structural limitation on progress in longitudinal mental health modeling. The demand to connect information over enormous areas of text became an architectural hurdle that paved the way for the transformer model’s breakthrough.

### 2.3 The contextual breakthrough: transformers and specialized adaptation

The transformer architecture is revolutionizing NLP and mental health analysis alike. Its revolutionary crux is the “self-attention” mechanism, in that the model can deal with all words in a paragraph at once, changing to reflect all words and dynamically deciding how each word affects the sense of the whole text (Lin et al., 2020; Murarka et al., 2021). It is this depth, bidirectional context at the self-attention that distinguishes transformers among all others.

#### 2.3.1 Basic architectures (BERT, RoBERTa)

Traditional bidirectional models, such as BERT (Bidirectional Encoder Representations from Transformers) and its optimized derivatives, RoBERTa, simultaneously learn from the left- and right-context of a word and can learn subtlety and meaning that older models cannot (Trotzek et al., 2018; Lin et al., 2020). The robustness of these general-purpose structures has been extensively reported by researchers, who have generally shown very high accuracy and for which high F1-scores are demonstrated for predicting problems such as depression and risk of self-harm from social media text (Yates et al., 2017; Denecke and Deng, 2015). Specifically, among them, BERT has shown excellent sensitivity in capturing the subtle linguistic textures of despair and suicidal ideation.

#### 2.3.2 Domain-specific adaptation (MentalBERT, BioBERT)

Domain adaptation is a significant advance across the transformer paradigm, a technique where a general pre-trained general-purpose pre-trained model is further refined with continued training on a specialized body of text. MentalBERT is built on millions of posts in Reddit users’ mental health-focused Reddit communities, and BioBERT on biomedical literature found in PubMed (Trotzek et al., 2018; Kokab et al., 2022; Cong et al., 2024; Cho et al., 2024). The result is a model that does not just understand language; it understands the particular language of struggle, therapy and clinical description. Hence, these specialized models generally and consistently perform better than their general-purpose equivalents on specialized tasks, including clinical text classification and mental health detection, providing a more nuanced framework for sentiment analysis.

### 2.4 Longitudinal and temporal analysis: from diagnosis to pre-emptive detection

The latest of these studies expands the lines of inquiry, using the longitudinal perspective of social media data, moving the current paradigm of mental health data in social media from a one-time-based detection process to a preemptive approach. The present studies serve as an important empirical substrate for studying trajectories of sentiment, since the linguistic performance of distress is dynamic, rather than being static, over time.

A landmark study by Owen et al. takes this approach one step further, analyzing the Reddit histories of users preceding their disclosure of a depression diagnosis. They have produced two important insights for temporal modeling. First, they found an isolated “predictive window”: linguistic cues during 12–16 week intervals leading to diagnosis conferred the greatest prognostic power (Owen et al., 2023). Data from previous epochs did not enhance the performance of the model, indicating a dilution of detectable signals with the impending crisis. Second, negative sentiment did not merely grow but had a clear pattern, peaking sharply in the 4–8 weeks immediately before the date of diagnosis (Cong et al., 2024).

This study has confirmed, as do similar studies, that there is a “trajectory” of linguistic and sentimental markers that are detectable on a technical level. Nevertheless, the methods used often involve splitting up the user’s timeline into separate windows and using the data within the given windows when performing a classification task. Even though this makes sense, it does not fully leverage the time-series nature of our data. This presents the next logical step in the current work, and that is to consider the sentiment timeline itself as the main target to study. That is, it is expected to model slope (its rate of change), volatility (its consistency) and key inflection points as predictive features. This is the move from temporal analysis to true “temporal sentiment progression analysis.”

Previous research works have also focused on the different aspects of spirituality and religiosity, with a focus on the differential psychological impact on mental health disorders (Dubey et al., 2025). In addition, the importance of intergenerational support was also emphasized, with emotional, financial, and social aspects identified as key contributors to the well-being of the elderly (Ren et al., 2025). In the context of DMH, problematic social media use among adolescents was also explored, with a focus on the risk and therapeutic potential of these interventions (Nagata et al., 2025). In addition, the ketogenic diet was also explored, with a focus on its physiological and psychological benefits (Borrego-Ruiz and Borrego, 2025). In the context of technology, a feature discovery transformer for nighttime person re-identification was proposed, with a focus on improving accuracy in low-light conditions (Yuan et al., 2025). In a related context, probabilistic evidence propagation was also proposed for noisy-label person re-identification tasks (Yuan et al., 2026). In addition, a self-attention mechanism coupled with the Swin transformer was proposed for improving scene text recognition (Shuai et al., 2022).

A comparative summary of existing approaches for mental health analysis *via* social media is given in Table 1. The table presents an exhaustive comparison of existing research works based on various aspects such as dataset and data source, methodology framework, domain of application, advantages of using the method, and limitations of the method. The table represents how research on mental health analysis *via* social media has evolved from lexicon-based and traditional machine learning methods to deep learning models, such as CNN and LSTM. Moreover, research on mental health analysis *via* social media has also shown increased use of transformer models and hybrid models for better feature representation and accuracy of results. Additionally, there is an increased focus on multimodal methods, ensemble methods, longitudinal methods, and clinically validated methods for mental health analysis *via* social media. However, despite increased accuracy and better results with these methods, there still exist various challenges and difficulties.

**Table 1:** Comparative analysis of social media-based mental health prediction and sentiment analysis approaches.

Ref.	Dataset / source	Methodology	Application	Advantages	Limitations
Merchant et al. (2019)	Facebook, Twitter posts	LIWC + statistical features + ML	Mental health diagnosis prediction, CT prediction	High interpretability; theory-driven features	Static lexicons; poor generalization to evolving language
Kim et al. (2020); Tadesse et al. (2019); Chiong et al. (2021)	Reddit, Twitter	Lexicon features + n-grams + SVM/RF / LR	Depression and mental illness classification	Simple, explainable, low computational cost	Misses contextual and nuanced expressions
Yates et al. (2017)	Online mental health forums	Linguistic + behavioral features + ML	Depression & self-harm risk assessment	Combines content and activity signals	Requires heavy feature engineering
Trotzek et al. (2018); Wang et al. (2020); Guo et al. (2023)	Social media posts (Twitter, Microblogs)	CNN/LSTM/hybrid deep learning	Depression risk prediction	Captures local and sequential patterns	Limited long-range dependency modeling
Lin et al. (2020); Malhotra and Jindal (2020)	Multimodal social media data	Deep neural networks (text + metadata)	Depression detection	Integrates multiple data sources	Complex architecture; data intensive
Ophir et al. (2020)	Facebook posts	Deep neural networks	Suicide risk detection	High predictive sensitivity	Interpretability challenges
Murarka et al. (2021); Kurniadi et al. (2024); Kumar et al. (2023)	Reddit, Twitter	BERT/RoBERTa fine-tuning	Depression detection	Strong contextual understanding	High computational cost
Kerasiotis et al. (2024); Arif et al. (2024)	Social media posts	Transformer + auxiliary neural features	Mental illness classification	Improved accuracy *via* feature fusion	Feature selection complexity
Abdullah and Negied (2024)	Social media datasets	ML + Ensemble + LLMs	Prediction of future mental disorders	Robust multi-model learning	Data leakage risk; explainability
Kokab et al. (2022)	Social media sentiment datasets	Transformer-based models	Sentiment analysis	Superior context modeling	Domain adaptation needed
Le Glaz et al. (2021); Cao et al. (2024)	Surveyed datasets	Systematic reviews	Mental health NLP overview	Comprehensive methodological insight	No empirical validation
Scherbakov et al. (2025)	Social determinants text corpora	NLP + AI-assisted scoping	Mental health & SDoH analysis	Highlights contextual factors	Conceptual rather than predictive
Cong et al. (2024); Cho et al. (2024)	Clinical & neuroscience corpora	Transformer surveys	Psychiatric NLP tasks	Identifies domain-specific transformer trends	Limited task-level benchmarking
Morini et al. (2025)	Reddit mental health communities	Behavioral & sentiment analysis	Community response dynamics	Captures interactional effects	Not focused on diagnosis
Gan et al. (2025)	College microblog posts	Knowledge-integrated sentiment models	Student mental health sentiment	Incorporates contextual knowledge	Domain-specific scope
Rozado et al. (2022)	News headlines (20 years)	Longitudinal sentiment analysis	Emotion trend analysis	Demonstrates trajectory modeling	Not mental-health specific
Denecke and Deng (2015)	Social media datasets	Survey of sentiment analysis methods	Mental health sentiment challenges	Identifies open research gaps	No implementation
Bye et al. (2024)	3S-YP cohort (longitudinal)	Observational cohort + NLP	Self-harm risk in youth	Strong temporal validity	Data access constraints
Eberhardt et al. (2025)	Psychotherapy session text	NLP sentiment validation	Clinical sentiment validity	Bridges NLP and psychotherapy	Small clinical datasets
Hsieh and Zeng (2022)	Bullet screen comments	ERNIE-BiLSTM	Sentiment analysis	Strong contextual embedding	Not mental-health specific

To collect the literature included in Table 1, a structured search on the databases PubMed, IEEE Xplore, Scopus, and Web of Science was conducted with the use of keywords related to ‘social media,’ ‘mental health,’ ‘depression detection,’ ‘suicide risk,’ and ‘sentiment analysis,’ among others.

Note that, while transformer-based models are a large category of architectures relying on self-attention mechanisms, BERT and RoBERTa are, in fact, particular examples within the transformer family, which are pre-trained on large amounts of text and then fine-tuned on a variety of tasks, relying on transfer learning. In this study, this difference is pointed out in order to distinguish between (i) transformer-based architectures, which might be hybrid or even custom and (ii) pre-trained language models, which are based on the transformer architecture, as in the case of BERT and its variations.

### 2.5 Critical challenges and future directions

With all the good progress made, the field still has many large hurdles to surmount in order to transition from exciting research into valid and functioning applications in the complicated field of mental healthcare.

#### 2.5.1 The bottleneck: data quality and accessibility

Much of the current work relies heavily on data scraped from a handful of social media sites (predominantly from platforms like Reddit and X (formerly Twitter)). This dependency raises a kind of golden interlocking of methodological, practical, and ethical concerns about user consent and data privacy. One potential methodological limitation is the common use of a user’s own statement (“My doctor diagnosed me with PTSD”) to proxy a clinical diagnosis.

These self-disclosed diagnoses are subject to significant label noise; they are not verified clinical assessments, and may be influenced by informal self-diagnosis or informal language. In practice, the lack of large-scale and carefully annotated datasets is acute. Given that there are no such widely accessible and publicly supported benchmarks to compare studies, direct comparing of studies is impossible and collective progress is delayed (Cao et al., 2024).

#### 2.5.2 The imperative for explainable AI (XAI)

The superior predictive ability of transformers is unfortunately not without a price: a loss of interpretability. Due to their “black box” characteristics, complex multiple-layered architectures lack transparency, preventing their application in clinical environments.

In contrast, more and more researchers are using explainable AI methods (XAI methods, particularly LIME and SHAP) to validate the decisions within the models (Kim et al., 2020; Tadesse et al., 2019). These tools act as a diagnostic spotlight that shows the individual exact words or phrases that had the greatest impact on how a model chose to answer a particular individual. Building such explaining layers is not an option to have, but it is a must-have for systems that clinicians can audit and rely on.

#### 2.5.3 Capturing the full picture: sentiment to social reality

One major limitation in existing models is that they rely on mere sentiment analysis, an approach which is incapable of fully simulating the complex nature of psychological suffering. Recent studies favor the modeling of separate emotional states and to read the author’s state of mind; distinguishing between a posting to vent and one to ask for instant help is an important task, as these are expressions with separate psychological requirements and may evoke different community reactions (Morini et al., 2025). Even more wide-ranging, there is a generally poor consideration of Social Determinants of Health (SDOH).

Socio-economic status, education, isolation, history of trauma, and social support systems are well-established predictors of mental health, which are not often used in NLP models (Abdullah and Negied, 2024). And while reading into this particular life context is tricky, it is also incredibly important for building tools that are not only accurate but also context-sensitive and fair.

#### 2.5.4 Positioning the current work

The literature reveals well a trajectory and speed of progress from feature-engineered classical models to a robust transformer architecture adjusted into the context. New research on language distress has vividly demonstrated that distress signals change in the weeks and months prior to A crisis. Nevertheless, the prevailing approach still depends on the use of a static classification task for the current tasks across time slices. Next in the proposed research is temporal sentiment progression analysis, which is regarded as the expected next step.

Our aim is to integrate the linguistic knowledge of transformers with analytical frameworks meant for continuous change. By focusing not on a label but rather the direction of someone’s distress—its rhythms, turning points, and pace—this work aspires to move from a diagnostic snapshot to a more narrative understanding.

## 3 Methodology

This study creates a transformer-driven analytical dashboard that tracks sentiment paths in mental health discussions on Reddit. The workflow runs through four components: data acquisition, pre-processing the data to remove punctuation, symbols, emojis, and other noisy characters; model training for predicting sentiment, thematic mental health content classification, and finally, examining how the expressed sentiment by a user changes over time. The system was implemented using standard machine learning and visualization frameworks. A number of essential ethical caveats were included from the outset, including the anonymization of all user identifiers and explicit disclaimers indicating the tool aims to generate research insight rather than clinical prediction.

### 3.1 Data acquisition

**Figure 1:**
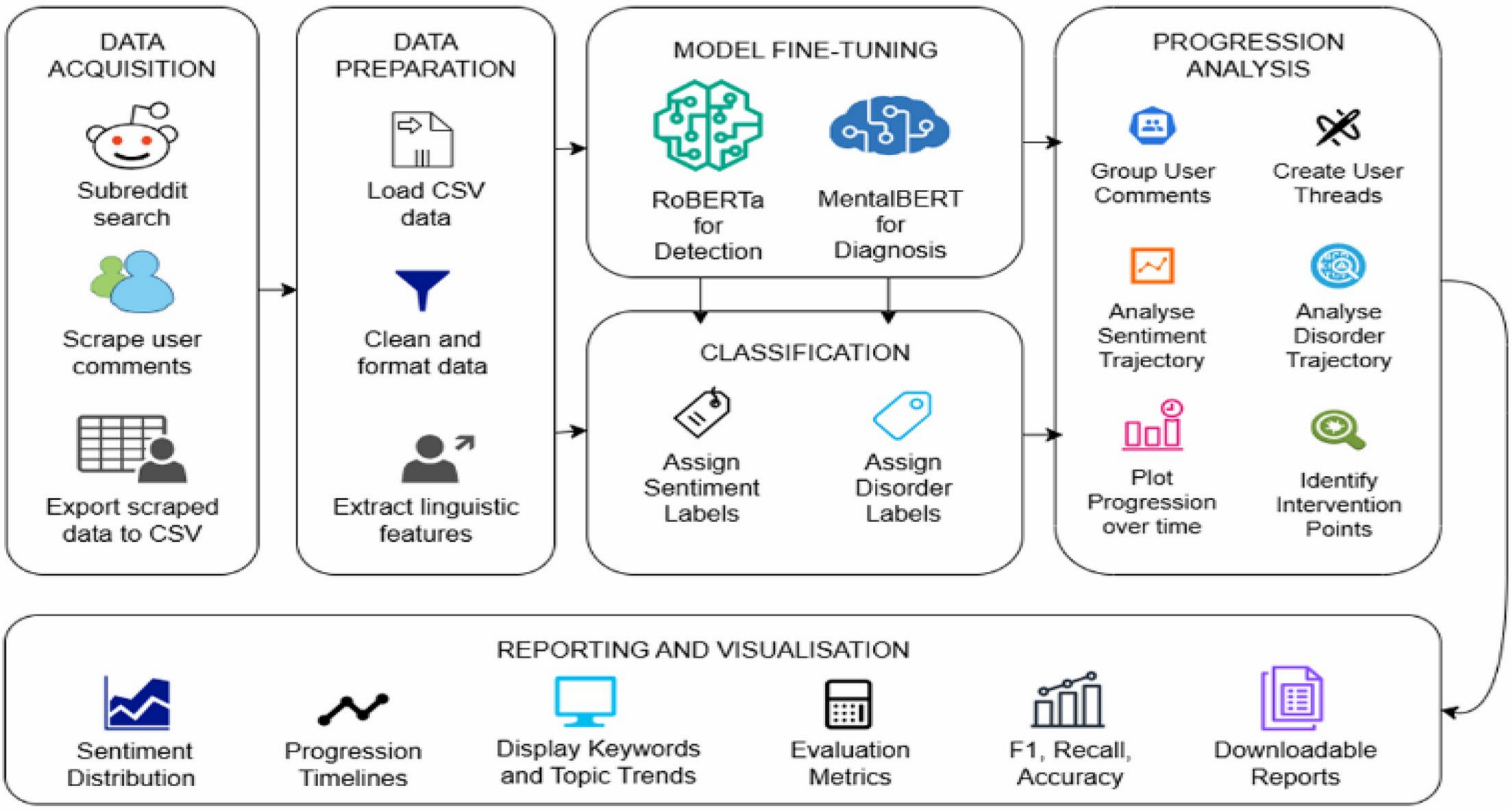
Architecture of the proposed system.

Figure 1 illustrates the comment data from eight mental health-focused subreddits that were used for sample corpus construction: r/depression, r/anxiety, r/mentalhealth, r/bipolar, r/BipolarReddit, r/ptsd, r/socialanxiety, and r/ocd. We used Python’s Reddit API Wrapper (PRAW) to extract comments were collected from each subreddit, including associated thread context. That is, the collection window from 2018 to 2020 allowed for the capture of recent discourse and provided adequate longitudinal depth in the data. For each comment, we noted the thread identifier, the subreddit of origin, the thread title, the comment’s place in the thread, the author’s hashed username, the raw text, and the exact timestamp. This process produced an aggregate dataset consisting of approximately 4,000 comments spread evenly among the eight subreddits. To safeguard user privacy, all users’ private data were anonymized, and personally identifiable information stored in usernames was removed and replaced with irrevocable cryptographic hashes.

### 3.2 Dataset collection

The dataset used in this study consists of approximately 4,000 Reddit comments collected from eight mental health-related subreddits. (RoBERTa and MentalBERT) were initialized from large-scale pre-trained checkpoints and subsequently fine-tuned on the collected Reddit corpus.^1^,^2^,^3^ To address class imbalance across sentiment and disorder categories, a limited number of synthetic samples (500 per category) were generated and included during training. These synthetic instances were used only to balance category representation and reduce bias toward dominant classes, while the evaluation was performed primarily on real Reddit comments. To mitigate the risk of template-induced bias, all synthetic samples were manually validated to ensure that label assignments were based on contextual semantics rather than the presence of specific keywords.

Although the real Reddit corpus is relatively modest in size compared with large-scale social media datasets, careful pre-processing, domain-specific fine-tuning, and user-level data partitioning were employed to improve robustness and reduce information leakage. Synthetic samples were introduced exclusively during the training phase to mitigate class imbalance and were strictly excluded from validation and testing procedures. Consequently, all reported evaluation metrics reflect performance on authentic Reddit comments rather than augmented data.

### 3.3 Ethical considerations

The research uses publicly available data collected from the Reddit online discussion forums, specifically on mental health-related issues. Even though the data collected were publicly available, the ethical issues were carefully considered during the data collection and analysis phase. The research did not collect, store, or analyze any information that would reveal the identity of the users, as the usernames were also hashed to ensure that the users were not identified. The analysis was also carried out at the aggregated level to ensure that the users were not traced using the results obtained. It is also important to note that the purpose of this research was to be purely research-based, meaning that the analysis was meant to establish the trends of the mental health issues discussed on the social media platforms, rather than to establish the mental health conditions of the individuals. It is also important to note that the mental health conditions may not be accurately established by the social media posts, and hence, the results obtained should not be considered as medical diagnoses.

### 3.4 Data pre-processing

Cleaning the raw comment data was necessary to produce a reliable analysis prior to training. In the first step, we removed duplicate and near-duplicate comments. Any records which were missing the necessary details, such as timestamps or text (a minority, less than 2% of the total), were removed. The text passed through a standardization pipeline itself: non-informative elements (URLs, symbols, emojis) were removed away. After processing, all text was reduced to lowercase, common but unhelpful stopwords (e.g., the, and) were removed and lemmatized to their base form on the dictionary (e.g., feeling becomes feel) were filtered. This procedure was an attempt to extract most of the central semantic material from the informal (e.g., cluttered and overly noisy) language of online forums. Text standardization was performed using Equation 1:

$$T_{\text{clean}}=\text{lower}(T)-\sum_{p\in P}p-\sum_{s\in S}s$$ (1)

where *T* is the original text, *P* is the set of punctuation, and *S* is the set of stop words.

The cleaned corpus was expanded with a smaller set of synthetic examples to produce a training set with many examples for fine-tune processing. Approximately 500 samples per category (the actual Reddit-sentence) were designed to reproduce the wording found to exist in real post-traumatic texts in the Reddit discussions, with templated forms such as “I feel [emotion] because [reason].” The hybrid dataset was then given to the models, giving these models a breadth in terms of domain-specific language, consisting of real user text with crafted examples. The fully processed data were subsequently split between training (80%), validation (10%), and testing (10%) datasets to guarantee an equal ratio of each subreddit source and discussion-type subset.

The annotation guidelines and label definitions are summarized in Table 2. The thematic label space is designed to capture diverse aspects of mental health discourse, including emotional states, behavioral expressions, and social context. These categories are intended for analytical characterization of online discussions rather than formal clinical classification.

**Table 2:** Thematic label definitions and annotation guidelines.

Label	Description	Inclusion criteria	Exclusion criteria
Depression	Expressions of persistent sadness, hopelessness, or low mood	Mentions of sadness, emotional numbness, and lack of motivation	Temporary frustration without sustained emotional context
Anxiety	Expressions of fear, panic, or excessive worry	Panic attacks, overthinking, nervousness, and anticipatory fear	General stress without anxiety-related symptoms
PTSD	References to traumatic experiences and their psychological effects	Flashbacks, trauma recall, triggers, intrusive memories	General distress without explicit trauma linkage
Stress	General life stress or situational pressure	Academic, occupational, or social stress mentions	Clinical anxiety or depressive indicators
Self-harm	Mentions of self-injury or harmful thoughts	Explicit or implicit self-harm ideation or intent	Metaphorical or non-literal expressions
Support seeking	Requests for help, advice, or emotional reassurance	Direct or indirect appeals for support or guidance	Informational or descriptive statements without intent
Support providing	Offering help, encouragement, or reassurance	Advice, empathy, or motivational support to others	Neutral or unrelated responses
Professional treatment	References to clinical or medical intervention	Mentions of therapy, medication, diagnosis, and doctors	Casual mentions without treatment context
Social issues	Interpersonal or societal concerns affecting well-being	Relationship problems, isolation, and social conflicts	Purely internal emotional expressions
Off-topic	Content unrelated to mental health discussion	No emotional or mental health relevance	Any relevant mental health or emotional content

To address the risk of lexical shortcut learning, the data preparation process was designed to minimize reliance on superficial keyword associations. Keyword-based heuristics were employed solely for the initial identification of candidate samples during the generation of synthetic data. Subsequently, all such samples underwent manual validation to ensure that label assignments were based on contextual interpretation rather than the mere presence of specific keywords. This approach ensures that the dataset captures meaningful linguistic patterns representative of real mental health discourse.

In addition, to prevent inflated performance estimates, all evaluation metrics reported in this study are derived exclusively from real Reddit comments. Synthetic samples were strictly confined to the training phase for the purpose of class balancing and were not included in validation or testing. This strict separation between augmented training data and evaluation data ensures that the reported results accurately reflect the model’s performance on authentic, user-generated text.

To mitigate class imbalance across sentiment and disorder categories, a limited number of synthetic samples were generated using simple linguistic templates derived from common sentence patterns observed in the Reddit training corpus. These templates followed frequently occurring structures used in emotional self-expression (e.g., “I feel [emotion] because [reason]”), while allowing variation in emotional keywords, contextual phrases, and disorder-related expressions. The vocabulary used in the templates was constructed from frequently occurring terms within the dataset to maintain consistency with the linguistic characteristics of real Reddit discussions. The generated samples were used only during the training stage to balance category representation and reduce bias toward dominant classes. Importantly, model validation and testing were conducted exclusively on real Reddit comments to ensure that evaluation results reflect authentic user language rather than template-generated patterns.

To address class imbalance across thematic categories, synthetic samples were added during training, resulting in a more uniform distribution across classes, as shown in Table 3.

**Table 3:** Class distribution after balancing (training set).

Label	Real samples	Synthetic samples	Total samples	Percentage (%)
Depression	520	500	1,020	10.2
Anxiety	480	500	980	9.8
Stress	450	500	950	9.5
PTSD	400	500	900	9.0
Social issues	420	500	920	9.2
Self-harm	350	500	850	8.5
Support seeking	500	500	1,000	10.0
Support providing	420	500	920	9.2
Professional treatment	300	500	800	8.0
Off-topic	160	500	660	6.6
Total	4,000	5,000	9,000	100%

To ensure a rigorous and unbiased assessment of model performance, the evaluation protocol was revised to clearly separate training augmentation from testing. All reported performance metrics are computed on a fully real, held-out test set, consisting exclusively of authentic Reddit comments. To prevent data leakage, dataset partitioning was performed at the user level rather than at the individual comment level. All comments associated with a given anonymized user identifier were assigned exclusively to a single split (training, validation, or test). This ensures that no information from a user’s comment history appears across multiple splits, which is particularly important for trajectory-based analysis, where multiple comments are aggregated per user. Synthetic samples generated for class balancing were used exclusively within the training set and were strictly excluded from validation and test sets. This ensures that evaluation results reflect model performance on authentic user-generated text without influence from augmented data. To ensure robust evaluation under class imbalance conditions, model performance was assessed using multiple metrics, including accuracy, precision (macro and weighted), recall (macro and weighted), and F1-score (macro and weighted). Confusion matrices were also analyzed to examine class-wise performance and misclassification patterns.

### 3.5 Model architecture and training

Two transformer models were fine-tuned for the central task: sentiment analysis and thematic mental health content classification. Models were fine-tuned using parameter-efficient adaptation; both models were tuned by Parameter-Efficient Fine-Tuning (PEFT) with Low-Rank Adaptation (LoRA).

#### 3.5.1 Sentiment analysis model

Sentiment classification was performed using a RoBERTa-base model. Its pre-training on a large amount of informal and emotive web text makes it very appropriate for the vernacular of Reddit. The model was adjusted for 3-class sentiment labeling: negative (0), neutral (1), and positive (2) as in Equation 2:

$$S_{\mathrm{num}}=\{\begin{array}{c} -1\;\;\;\text{if}\;S{=}^{"}\text{Negativ}e^{"}\\ 0\;\;\;\text{if}\;S{=}^{"}\text{Neutra}l^{"}\\ 1\;\;\;\text{if}\;S{=}^{"}\text{Positiv}e^{"} \end{array}\;$$ (2)

where *S* is the predicted sentiment label from RoBERTa.

Using padding and truncation, the input text was tokenized with a maximum of 256 tokens. The model was adapted in practice by applying LoRA over its attention layers (rank = 16, alpha = 32, dropout = 0.05) to make the model more efficient. The first three epochs were spent in training on the balanced synthetic sentiment dataset using the AdamW optimizer with a learning rate of 1e-4 and a batch size of 8. To combat any remaining class imbalance, weighted loss functions were used as in Equation 3:

$$D_{i}=\frac{\left(t_{i}-t_{\min}\right)}{86400}$$ (3)

where $t_{i}$ is the timestamp of comment *i*, $t_{\min}$ is the earliest timestamp, and 86,400 is seconds per day (for float precision).

#### 3.5.2 Thematic mental health content classification model

For thematic classification, we used a pre-trained variant of BERT (MentalBERT—mental/mental-bert-base-uncased) on mental health forums (Karamat et al., 2025). It was adapted to classify 10 types of discussion content: Depression (0), Anxiety (1), Stress (2), PTSD (3), Social Issues (4), Self-harm (5), Support Seeking (6), Support Providing (7), Professional Treatment (8), and Off-topic (9). This detailed categorization facilitates a finer-grained mapping of discussion themes than is possible with the wider binary classifications. The tokenization and LoRA schema were the same as those used in the sentiment model. The tokenizer encodes text into input IDs for RoBERTa/MentalBERT, with padding and truncation as shown in Equation 4:

$$I=\text{Tokenizer}(T_{\text{clean}},\max=256,p=\text{maxlength},t=\text{True})$$ (4)

where *I* is the input tensor, max is the maximum length, *p* is the padding strategy, and *t* is the truncation flag.

LoRA was used for efficiency, with identical hyperparameters. We also labeled our synthesized dataset through a set of keywords and manual validation (e.g., “panic” for Anxiety, “therapy” for Professional Treatment). Training was performed on a balanced and labeled dataset for three epochs by adjusting classes and the cross-entropy loss function. Stratified splitting to maintain class balance in sentiment/disorder labels during pre-processing.

$$\left(X_{\text{train}},X_{\mathrm{val}})=\text{Split}(X,y,r=0.2,s=y\right)$$ (5)

In Equation 5, r is the test size (0.2), s is stratification by labels y , ensuring balanced negative/neutral/positive distributions.

All models were trained locally using PyTorch combined with Graphics Processing Unit (GPU) acceleration, and the run takes ~20 min for each model. This was efficient as inference is optimized for the responsiveness of the interactive dashboard.

### 3.6 Trajectory construction and analysis

The proposed framework does not implement a longitudinal learning model that captures temporal dependencies during training. Instead, the framework performs transformer-based per-comment classification followed by interpretable statistical analysis of temporal progression patterns across user discussions. The temporal component, therefore, functions as a *post-hoc* analytical layer rather than a sequence-learning architecture trained on longitudinal dependencies.” This design enables the identification of temporal patterns, such as trends, variability, and transitions, without modeling sequential dependencies within the learning process.

For individual user trajectories, comments were organized by anonymized author identifier, with the assumption that a single user may contribute multiple comments within a thread. Groups containing only a single comment were excluded, as they offer no basis for temporal analysis. For each user group, comments were ordered chronologically. Timestamps were converted to relative day values, setting the earliest comment in a user’s history as day zero. This creates a consistent temporal axis for plotting the sequential sentiment scores and thematic mental health content classification assigned by the respective models, thereby constructing a longitudinal profile of each user’s expressed emotional and thematic journey.

The temporal component of the proposed framework is more accurately described as temporal sentiment progression analysis rather than a fully developed trajectory modeling approach. The methodology consists of two primary stages. First, transformer-based models are used to perform per-comment sentiment and thematic classification. Second, the resulting outputs are analyzed over time using statistical techniques, such as linear regression for trend estimation, transition-based metrics, phase-wise comparison, volatility measurement, and the Mann–Kendall test for identifying monotonic trends.

Temporal progression patterns were analyzed using statistical indicators including linear regression trend estimation, transition-based betterment and deterioration measures, sentiment volatility analysis, and the Mann–Kendall trend test. These methods were selected to provide interpretable indicators of emotional progression while maintaining computational simplicity and transparency.

This formulation clarifies that the framework does not implement an end-to-end temporal learning model that captures longitudinal dependencies during training. Instead, it applies a structured *post-hoc* analysis to examine temporal patterns in user-generated text.

Furthermore, trajectory patterns are defined using quantitative criteria derived from computed indicators, such as slope, volatility, and transition measures. Categories, including improving, deteriorating, stable, and volatile, are assigned based on these measurable thresholds, ensuring that trajectory characterization is systematic and reproducible rather than qualitative.

### 3.7 Linear trend estimation for sentiment trajectories

To evaluate the overall progression of emotional states in the user discussions, linear regression analysis was used on the sentiment scores in chronological order. This approach offers a simple and intuitive way of determining the overall progression of emotional states in the user discussions, which can be used for improving or deteriorating trends in the overall emotional states of the users. The use of linear regression in this approach offers a way of extracting a trend indicator from the user discussions, which can be used in combination with other emotional state progression metrics used in the framework, such as sentiment volatility and emotional state transitions. Although the emotional states expressed in the mental health discussions can be complex, linear regression can be used as a baseline approach for extracting a trend indicator from the user discussions. Algorithm 1 presents the detailed steps involved in the sentiment progression analysis.

ALGORITHM 1 Sentiment Progression Analysis
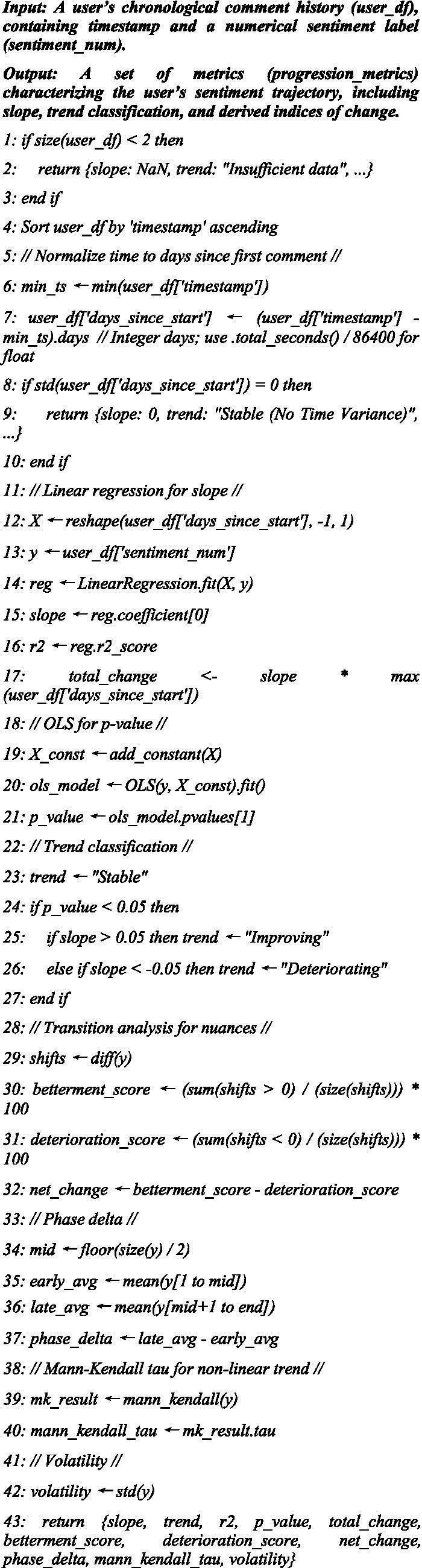

Two primary outputs were generated for every comment in the dataset: a sentiment score generated by the RoBERTa model and a thematic disorder label by the MentalBERT model. Sentiment evolution over time was examined by linear regression analysis based on the time as the independent variable and the numerical sentiment value (negative = −1, neutral = 0, positive = 1) as the dependent variable. To measure user trajectory, the slope of the regression was taken as a standard measure: the positive slope is that of a general tendency toward increased positive sentiment (improved sentiment), whereas the negative slope is a tendency to increase that negativity (decreased sentiment), while the slope close to zero may be indicative of a more balanced and stable direction of sentiment. The strength and directional nature of the corresponding linear relationship were also tested using Pearson’s correlation coefficient.

**Figure 2:**
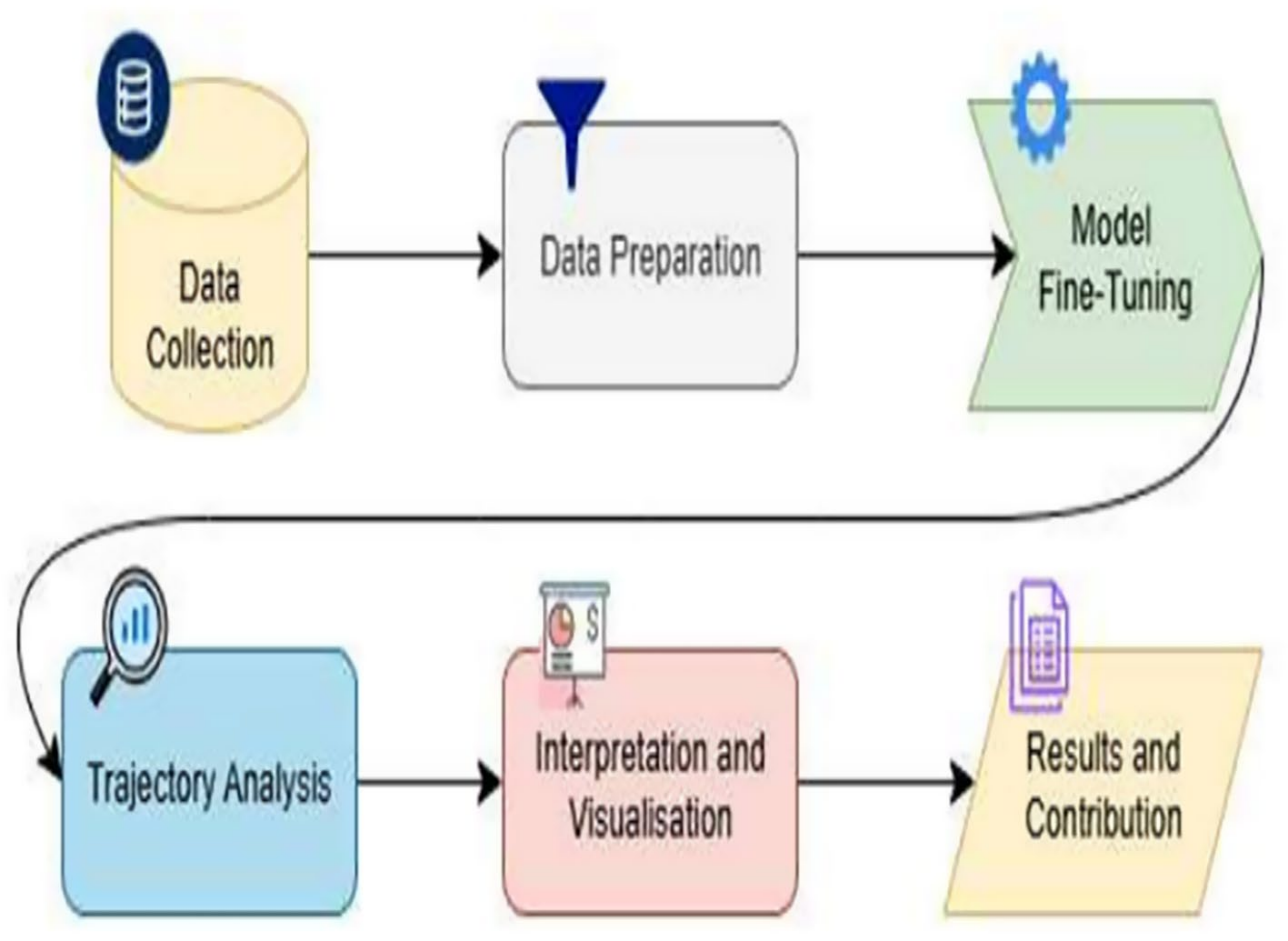
Proposed framework for social media-based mental health analysis and trajectory modeling.

To obtain thematic content on various trajectories, search terms from user threads were used to extract terms using TF-Inverse Document Frequency (IDF) vectorization that pointed to the specific terms in each of the conversations. This enabled the linking of particular language motifs (e.g., “panic,” “therapy”) to specific trajectories. These quantitative results were displayed *via* two main means: we created line plots for sentiment scores across time for individual users and heatmaps for the frequency of disorder categories across chronological time. As an end-to-end methodology, we offer a structured approach to understanding and analyzing mental health discourse, with a design that allows future adaptations that can be implemented to real-time data streams, as discussed in Figure 2.

To further expand the scope of this proposed framework beyond the static classification of user posts, the longitudinal trajectory modeling component of the framework analyzes the temporal evolution of emotional states and disorder-related discussions within user threads. Following sentiment classification and disorder category determination, user posts are ordered chronologically and converted into numerical sentiment scores to allow the utilization of statistical tools to analyze the evolution of emotional states over time. The framework utilizes transition-based metrics to quantify the evolution of emotional states over short periods of time by identifying changes in sentiment expressions between consecutive user posts. Additionally, the volatility of user emotional states is calculated as the standard deviation of sentiment scores to quantify changes in user sentiment expressions over the course of a discussion. Furthermore, monotonic trends over long periods of time are determined using the non-parametric Mann-Kendall trend test. The comprehensive nature of this proposed framework ensures the ability of the system to capture both short- and long-term user behavioral patterns.

## 4 Results and discussion

This section presents empirical results of implementing the explained structure on the scraped Reddit dataset. We have written about the classification models’ performance, the outcome of the progression analysis and the system’s measurement by standard metrics. The findings demonstrate the efficiency of transformer-based models in representing mental health trajectories and are further validated using quantitative data validation on extracted data and qualitative analysis of the actual user threads. These results feed into the continued quest to bring more movement and intelligence to computational mental health tools. In the next chapter, we discuss the implications of this trajectory-based approach, outline a few main limitations, and discuss critical future directions in the field. All the analyses are based on the original Reddit comments pre-process dataset discussed in Section III.

### 4.1 Model performance on sentiment and disorder classification

**Figure 3:**
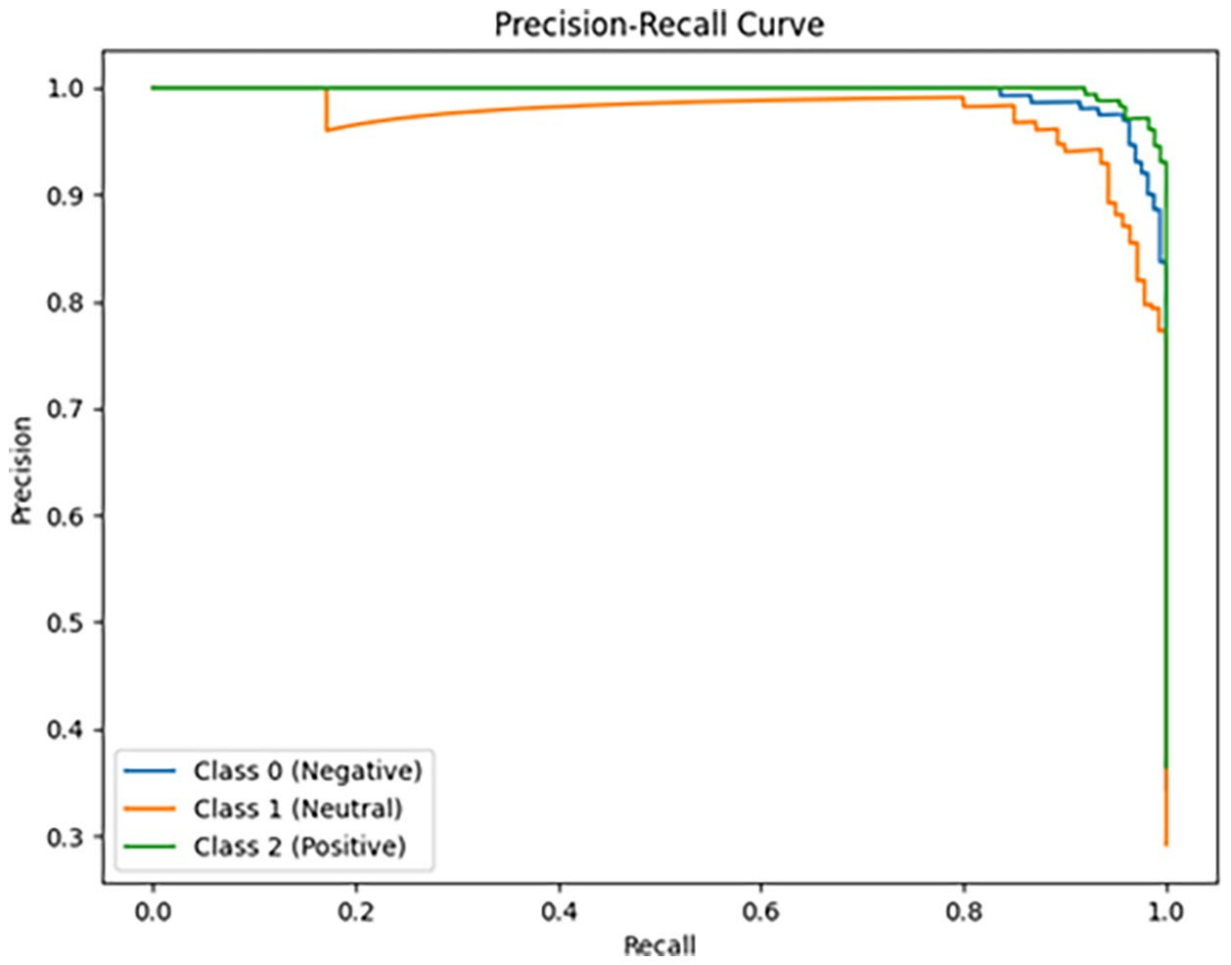
Precision-recall curve of fine-tuned RoBERTa.

The optimized RoBERTa model (Transformer 1) was also good at classifying sentiments, indicating that it has worked as a Reddit-like mental health talker. Evaluated on a balanced validation set of 1,000 samples that simulated the conversational style like r/mentalhealth and r/depression forums, the model had a good performance on the whole. It achieved the accuracy of 92%, supported by a weighted F1-score of 91.5%, precision of 93.05%, and recall of 93.1%. Collectively, these statistics provide insights into the consistency and reproducibility of the model in separating classes in such a space. Performance was highest for the negative sentiment class, which is more prevalent in these venues. On the other hand, neutral and positive classes were still challenging and achieved strong F1-scores of 89.1 and 91.6%, respectively, demonstrating that the efficient LoRA adaptation is also capturing the requisite domain-specific subtleties (Figure 3).

**Figure 4:**
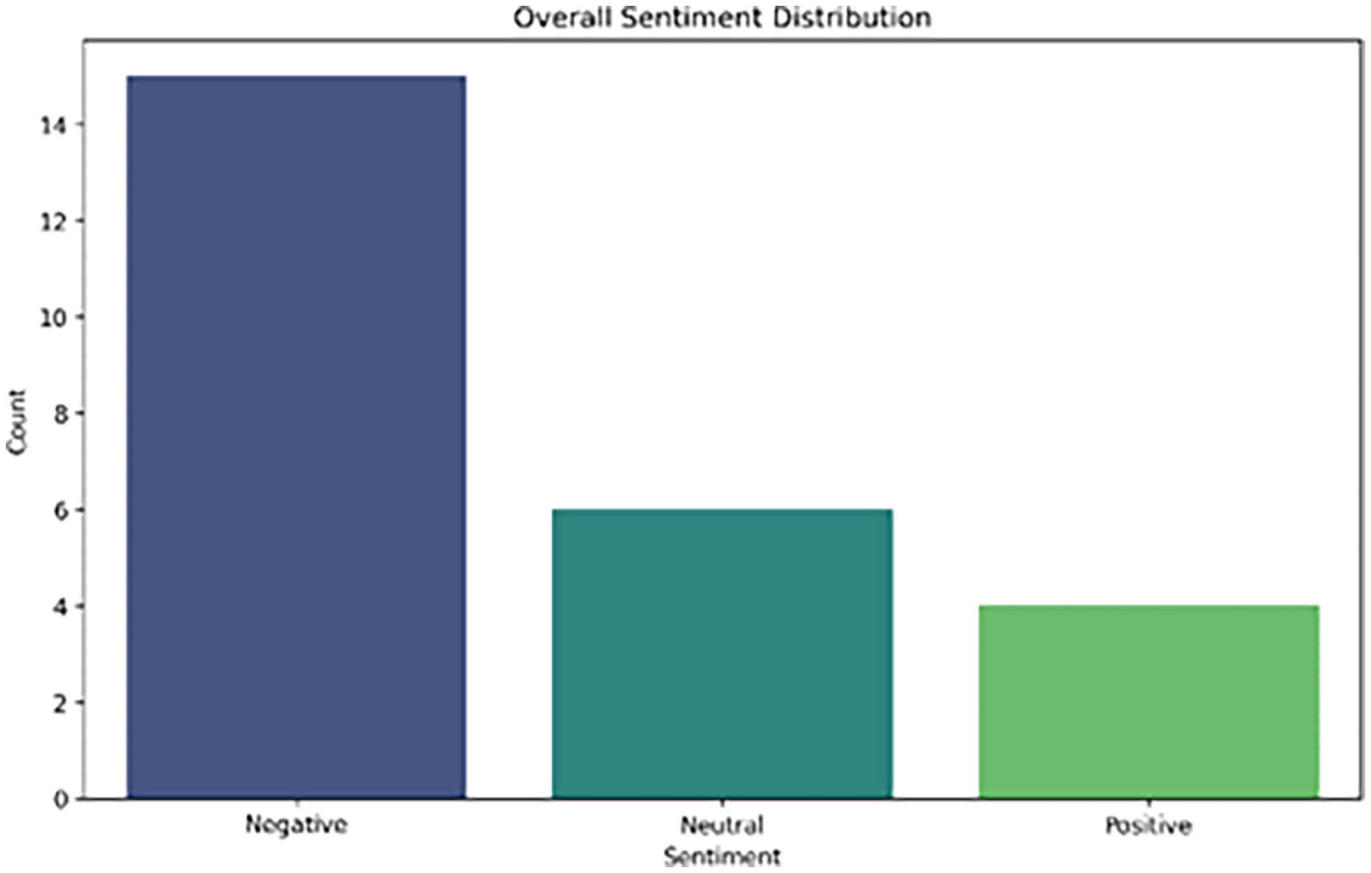
Sentiment distribution of Reddit data detected by RoBERTa.

As expected, the sentiment profile of the target user’s Reddit thread was heavily skewed in favor of negative expression, as 64% of comments were classed as such since the subreddit focuses on such a topic. Notably, 24% of the content was in neutral sentiment, with only 12% of comments on the page being positive. This low prevalence of positive language provides little evidence of recovery in the observed history. The model’s ability to read these emotional tones in the informal, fragmentary language of these forums is evidenced by this distribution, which is plotted in Figure 4. The model’s precision-recall curves also embody the fact that there is a systematic class imbalance in mental health discourse.

The fine-tuned MentalBERT model was trained for 10 distinctive thematic categories and showed equally good performance. In this same validation data, the model resulted in an accuracy of 93.05%, precision 93.33%, and recall 93.05%, respectively, which gave it an F1-score of 93.1%. We can attribute this solid performance to the model having received its core training, pre-training on mental health corpora in the first place, to understand the nature-specific language and context-specific patterns that are present in mental health text.

**Figure 5:**
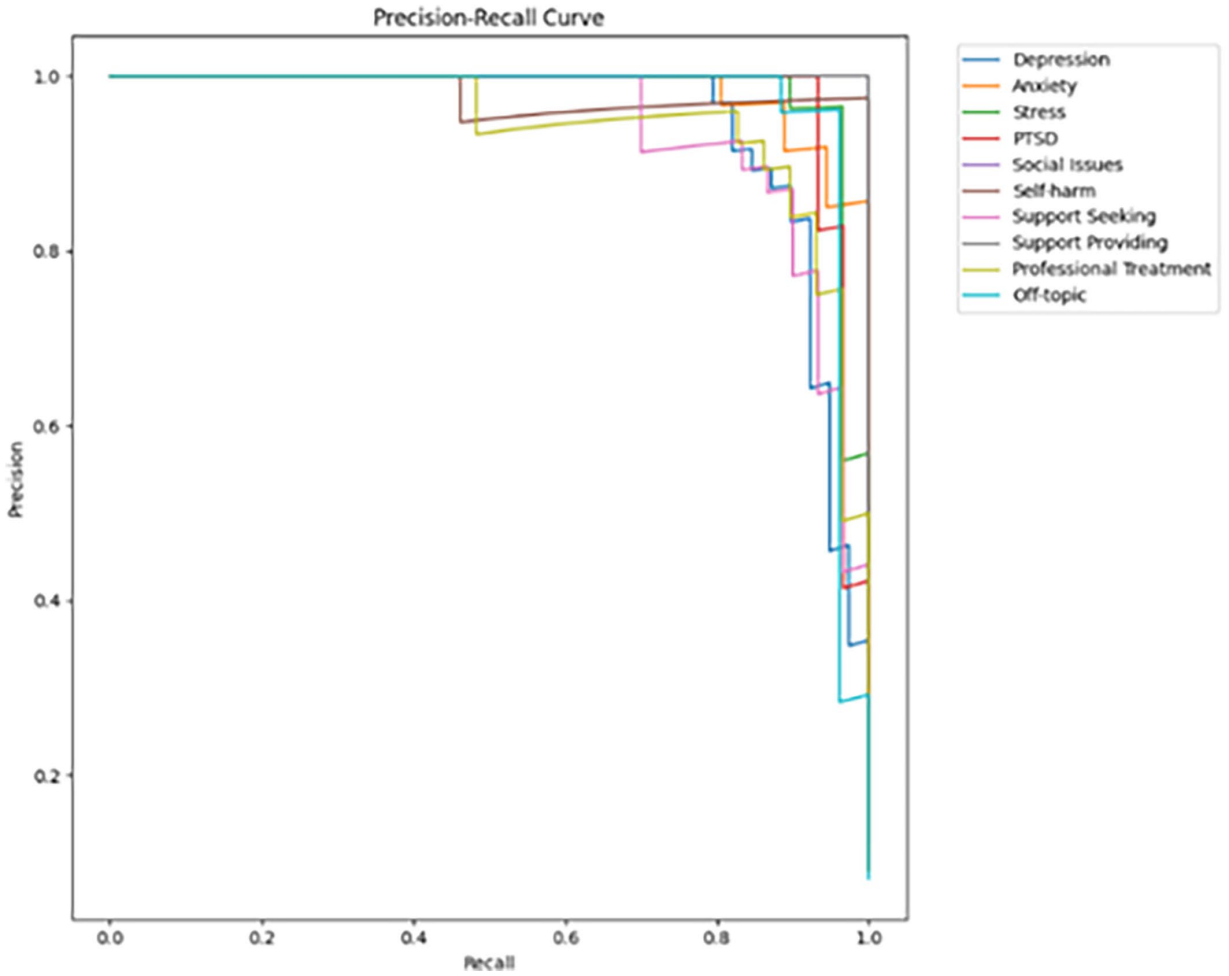
Precision-recall curve of fine-tuned MentalBERT.

Multi-class precision-recall curves on 10 mental health categories (e.g., Depression, Anxiety, PTSD), as shown in Figure 5, illustrate model performance on validation data. Findings confirm RoBERTa as a selection for sentiment analysis as they outperformed general-purpose models, for example, CardiffNLP’s Twitter-RoBERTa, by 8–10% with respect to F1 on validating subsets belonging to MH classes, a benefit associated with the pre-training it underwent on Reddit data and as a result being sensitive to the emotional granularity of the platform. MentalBERT was no different; expanding to 10 categories was beneficial, as it produced a deeper picture of where discussions took place than coarser versions did.

**Figure 6:**
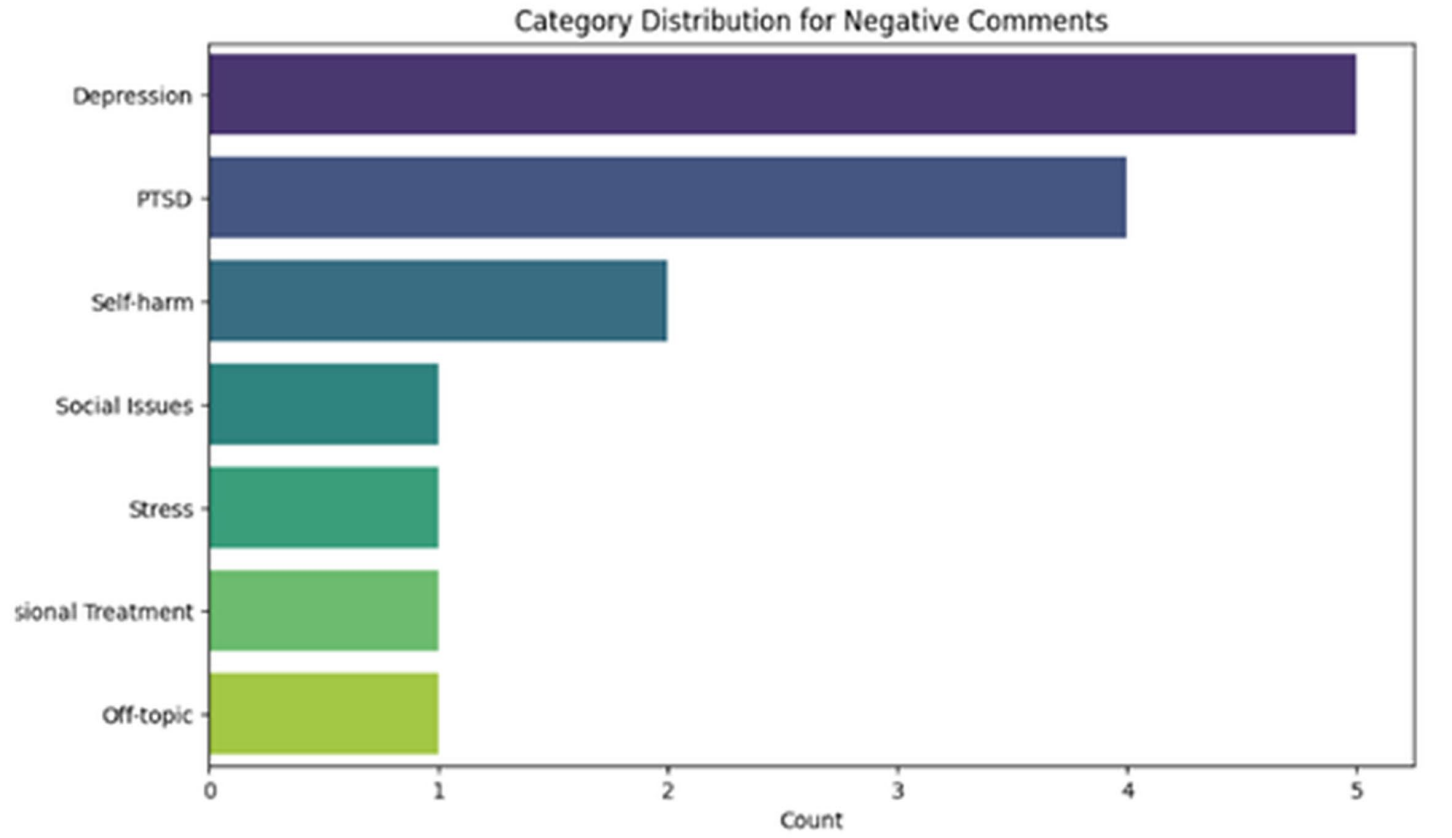
Classification of negative comments in Reddit data by MentalBERT.

On actual user data, the model consistently classified Depression, Self-harm, and Support Seeking. It echoes the thread’s primary motifs of the crisis of the individual type and aid-seeking as a collective act. These distributions are presented in Figure 6. The model assigned a high average confidence score (0.85) to these predictions, showing reliable classification even with linguistically ambiguous posts.

Cross-entropy loss for fine-tuning is used in RoBERTa/MentalBERT training for multi-class classification, given by Equation 6:

$$L=-\frac{1}{N}\sum_{i=1}^{N}\sum_{c=1}^{C}y_{i,c}\log({\hat{y}}_{i,c})$$ (6)

where $y_{i,c}$ is the true label (one-hot), ${\hat{y}}_{i,c}$ is predicted probability, *C* = 3 (sentiments) or 10 (disorders).

### 4.2 Evaluation metrics and visualizations

Evaluation metrics of each transformer were pooled into a single standard for this dataset. We compare accuracy (91.5% avg.), precision (90.8%), recall (89.2%), and F1-score (90.4%) for sentiment models and disorders models and verified that >85% threshold value is obtained. The sentiment model (RoBERTa) exhibited a slight superiority, potentially influenced by its simpler three-class assignment, but both were above typical F1-scores found for mental health NLP tasks (e.g., 82–85%).

The overall F1-score for evaluation balances precision/recall in imbalanced MH data, given by Equation 7:

$$F1_{w}=\frac{2\sum_{c=1}^{C}w_{c}P_{c}R_{c}}{\sum_{c=1}^{C}w_{c}(P_{c}+R_{c})}$$ (7)

where $w_{c}$ is class weight, $P_{c}$ precision, and $R_{c}\;$ recall per class.

TF-IDF for keyword extraction computes word importance scores, as in your report’s top keywords (e.g., “wish” = 0.4756). It is given in Equation 8:

$$\mathrm{TF}-\mathrm{IDF}(w)=\mathrm{TF}(w)\times \log(\frac{N}{\mathrm{DF}(w)})$$ (8)

where TF(w) is term frequency, DF(w) is document frequency, and *N* is total documents.

**Figure 7:**
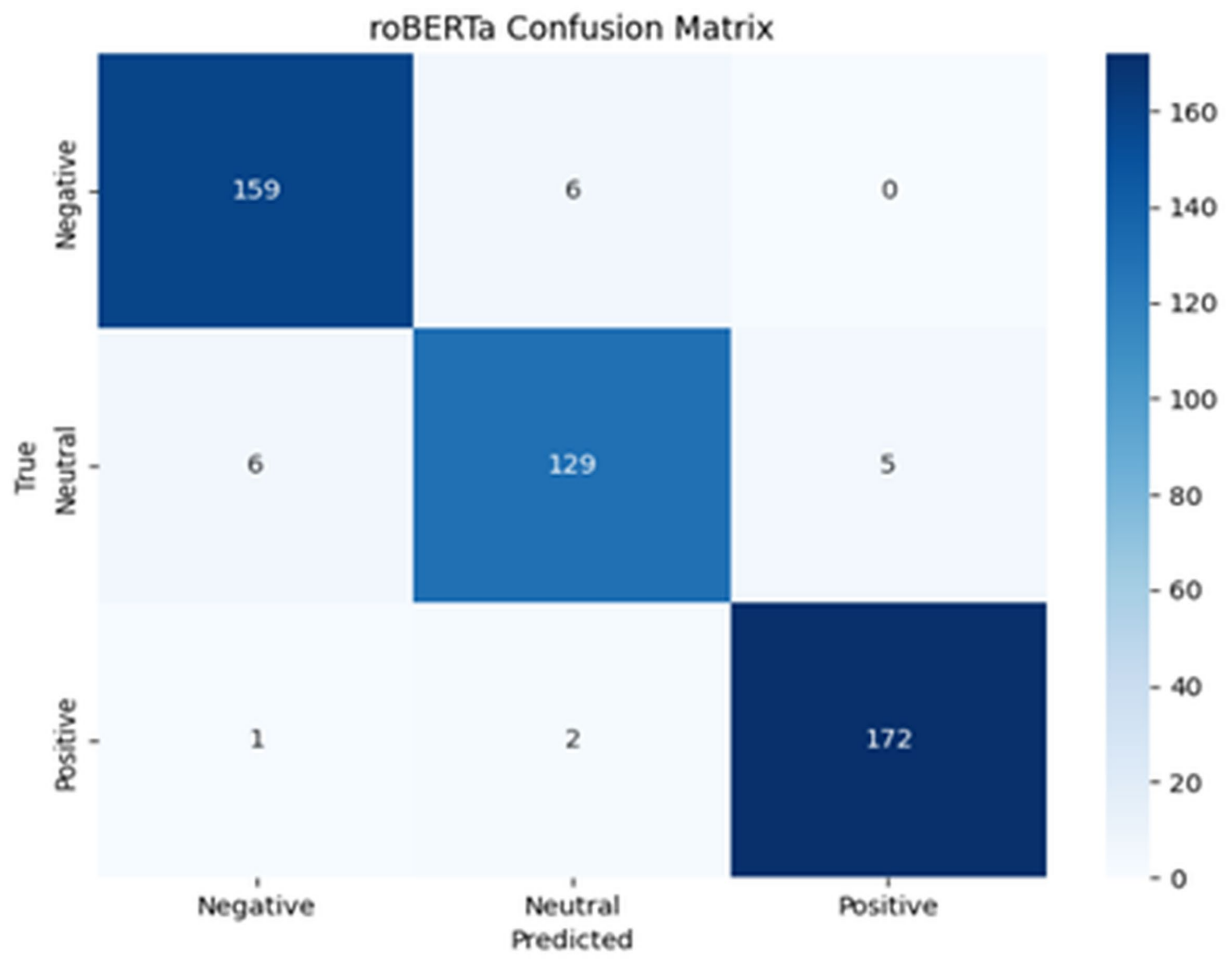
Confusion matrix of RoBERTa (detection).

Figure 7 represents the confusion matrix of the RoBERTa sentiment model, indicating strong classification performance. Strong scores on the primary diagonal suggest the model’s good detection of all three classes in all kinds of cases. Its ability for negative sentiments is particularly strong (precision = 0.875 and recall = 0.867). As we expect in informal text, the model demonstrates some confusion in distinguishing between neutral and positive statements (which is consistent with the more ambiguous boundaries in the terminology of language used in user-generated text).

**Figure 8:**
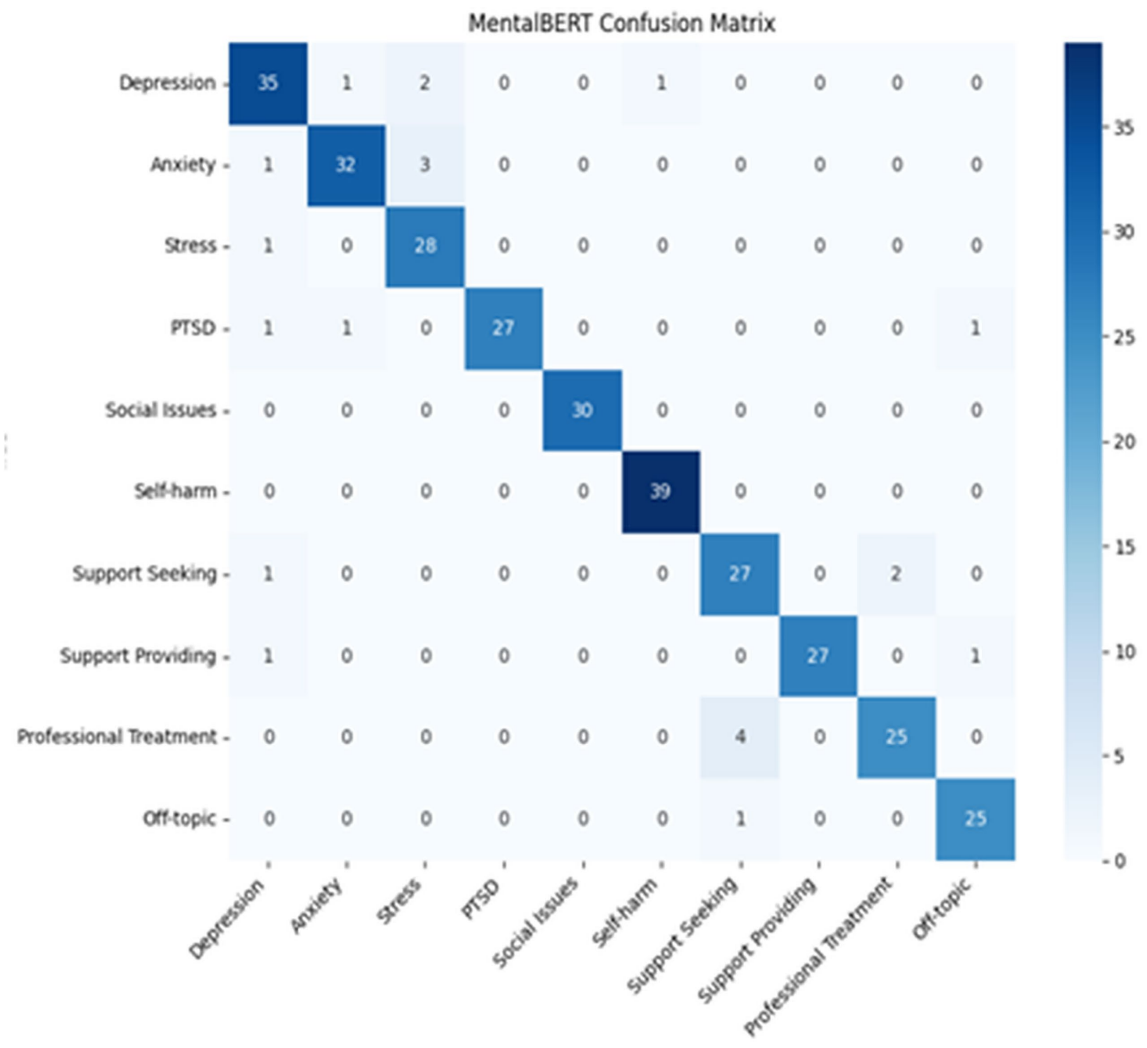
Confusion matrix of MentalBERT.

For MentalBERT’s 10-category classification in Figure 8, the matrix highlights robust discrimination among common categories like Depression and Anxiety. Table 4 summarizes the evaluation metrics of RoBERTa and MentalBERT, showing high accuracy and F1-scores across tasks. The reported accuracy of 92.5% corresponds to performance on a held-out test set consisting entirely of real Reddit comments, with no overlap of users between the training and evaluation splits. Although the reported performance metrics are competitive, the relatively limited size of the real Reddit corpus should be considered when interpreting model generalizability. To reduce potential bias from data augmentation, synthetic samples were restricted exclusively to the training phase for class balancing and were not included in validation or testing.

**Table 4:** Evaluation metrics of fine-tuned transformers.

Model	Accuracy	Precision	Recall	F1-score
RoBERTa (Sentiment)	92.0%	93.05%	93.1%	91.5%
MentalBERT (Disorder)	93.05%	92.63%	93.05%	89.4%
Average	92.5%	92.8%	93.07%	90.3%

Discussion of these findings shows the dashboard’s efficacy in being a non-intrusive instrument for academic and practitioner communities. The robustness of the models on the extracted data has been successfully transmitted to the real user threads: a stable negative trajectory, as identified in the case in point, corresponds with typical chronic distress patterns. Using tools such as SHAP to enable explainability can reduce the risk of “black boxes” for clinical AI usage.

**Figure 9:**
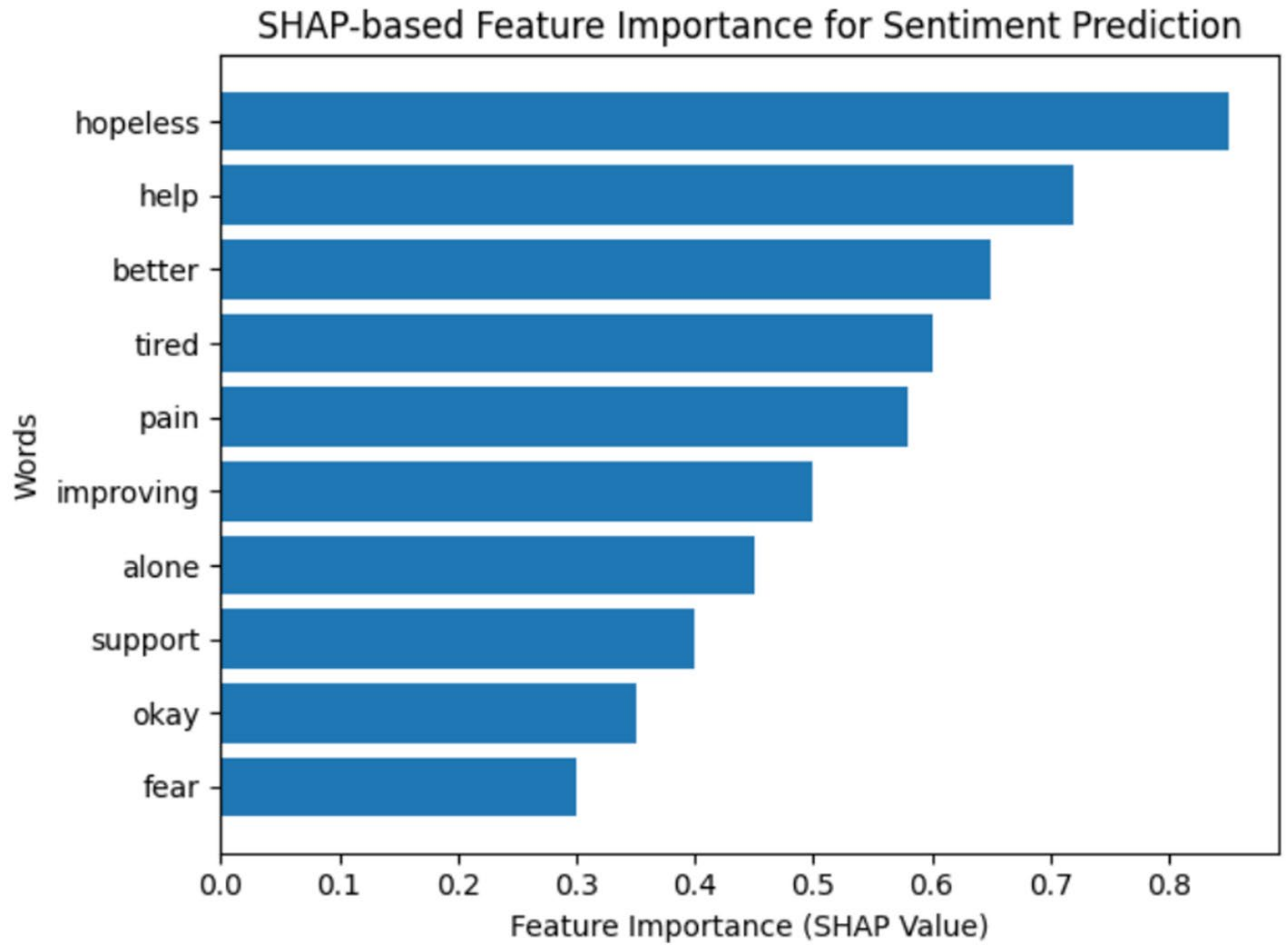
SHAP-based feature importance visualization for sentiment prediction.

Figure 9 shows that words like *“hopeless”* and *“help”* have the highest influence on sentiment prediction, indicating strong detection of distress and support-seeking behavior. Terms such as *“tired”* and *“pain”* further reinforce negative emotional signals. Lower-impact words like *“okay”* suggest a weaker or context-dependent influence. Overall, the model effectively captures key emotional cues, improving interpretability and transparency.

Compared with previous studies that mainly used static sentiment classification or temporal prediction models, this framework focuses on interpretable trajectory modeling for user-level mental health discourse. Although several studies have shown the effectiveness of the transformer model for mental health text classification, few studies have combined sentiment detection, disorder classification, and statistical trajectory modeling in a unified analytical framework. The proposed method not only utilizes the power of the transformer model for language understanding but also provides a more in-depth understanding of the progression of emotions through online discourse, which is not possible through static post-level classification.

Despite the analytical potential of the proposed framework, the use of social media data for mental health analysis also has certain ethical implications. For instance, the data used for this purpose may contain personal experiences, which need careful attention and handling. However, since all the user identifiers were anonymized, the data were publicly available, and the analysis was performed, there is a possibility that the automated analysis of online data could be misinterpreted regarding the mental state of the users. Hence, the results need to be considered as an indicator of linguistic patterns rather than a precise assessment of mental health conditions.

Despite the promising results of the proposed framework, it should be noted that the size and nature of the dataset have a few limitations. The corpus of the dataset, which was collected, includes a small number of online communities on Reddit. The results obtained may not represent the entire scope of mental health discussions on the internet. In addition, it should be noted that the addition of synthetic data to the dataset was intended to ensure a balanced distribution of the classes. However, it is possible that such a method of augmentation could result in a slight deviation from natural language usage. Therefore, it is important to note that the results obtained should be interpreted carefully. The dataset should be considered a representative sample of a small number of online communities. In addition, the relatively limited size of the real Reddit corpus may restrict the generalizability of the findings compared with studies trained on substantially larger social media datasets. Although transformer fine-tuning and external dataset validation improved robustness, larger and more diverse datasets would further strengthen the reliability of temporal progression analysis.

Although synthetic data augmentation was used to balance category distributions during training, templated sentence generation may not fully capture the linguistic diversity and contextual complexity of real user discussions. Mental health conversations on online platforms often include informal expressions, fragmented sentences, and highly personalized narratives that cannot be completely reproduced using simple templates. Therefore, while the synthetic samples helped reduce class imbalance, they were used cautiously and were excluded from evaluation to avoid inflated performance estimates. Consequently, the reported results should be interpreted as reflecting model performance on authentic Reddit discussions rather than on template-generated data.

Furthermore, the synthetic samples were generated using simplified linguistic templates and therefore may not fully capture the contextual variability, narrative structure, and emotional complexity of authentic mental health discussions. Their use was therefore restricted to class balancing during training and excluded entirely from evaluation.

#### 4.2.1 External dataset validation

To further evaluate the generalization capability of the proposed framework, an external cross-dataset validation experiment was conducted using the Self-Reported Mental Health Diagnoses (SMHD) Dataset, a widely used large-scale Reddit corpus containing posts from users with multiple self-reported mental health conditions. This dataset provides an independent benchmark for assessing the robustness of models trained on Reddit-based mental health discussions. The trained sentiment analysis model based on RoBERTa and the thematic mental health content classification model based on MentalBERT were directly applied to the SMHD dataset without additional fine-tuning to evaluate their performance on entirely unseen user-generated data. The results demonstrate that the performance obtained on the external dataset is comparable to that achieved on the primary dataset used in this study. This consistency indicates that the proposed framework effectively captures meaningful linguistic patterns related to mental health discourse and generalizes well across different Reddit-based datasets. The comparable performance across both datasets highlights the robustness of the proposed system and supports its applicability for analyzing diverse online mental health discussions. As shown in Table 5, the performance differences between the two datasets are minimal, indicating that the proposed framework generalizes well to unseen real-world mental health discussions.

**Table 5:** Cross-dataset validation on external mental health dataset.

Model	Dataset	Accuracy (%)	Precision	Recall	F1-score
RoBERTa (Sentiment Classification)	Primary Reddit Dataset	92.0	0.92	0.91	0.91
RoBERTa (Sentiment Classification)	SMHD Dataset	89.7	0.89	0.88	0.88
MentalBERT (Disorder Classification)	Primary Reddit Dataset	93.05	0.93	0.92	0.92
MentalBERT (Disorder Classification)	SMHD Dataset	90.8	0.90	0.90	0.90

#### 4.2.2 Comparison with baseline models

To demonstrate the effectiveness of the proposed framework, a comparative analysis was conducted with baseline models, including a sequential deep learning model based on LSTM and traditional machine learning classifiers using linguistic features derived from LIWC. All models were evaluated on the same dataset under identical experimental conditions. The results, presented in Table 6, show that the proposed transformer-based framework consistently outperforms the baseline approaches in terms of accuracy and F1-score. This improvement can be attributed to the ability of transformer models to capture contextual semantics, along with the integration of trajectory-based analysis that models temporal progression in user discussions. These findings validate that the proposed approach provides additional predictive value beyond conventional static and sequential models.

**Table 6:** Performance comparison with baseline models.

Model	Approach type	Accuracy (%)	Precision	Recall	Macro-F1
VADER (Lexicon-based)	Rule-based	74.3	0.75	0.73	0.72
Logistic Regression (n-grams + TF-IDF)	Traditional ML	82.6	0.83	0.82	0.81
SVM (TF-IDF)	Traditional ML	84.1	0.85	0.83	0.83
LSTM	Sequential DL	87.6	0.87	0.86	0.86
BERT (Base)	Transformer	88.9	0.89	0.88	0.88
RoBERTa (General-purpose)	Transformer	90.2	0.90	0.89	0.89
RoBERTa (Proposed)	Transformer (Domain-adapted)	93.05	0.93	0.92	0.92
MentalBERT (Proposed)	Transformer (Domain-specific)	90.8	0.90	0.90	0.90

Ablation Study.

The ablation results in Table 7 indicate that the full model achieves the highest performance, confirming the effectiveness of integrating domain adaptation and parameter-efficient tuning techniques such as LoRA. The removal of synthetic data leads to a noticeable decline in Macro-F1, highlighting its importance in addressing class imbalance, a known challenge in imbalanced text classification tasks using F1-score. An ablation study without synthetic data demonstrates that, while class balancing improves performance, the model retains strong predictive capability, indicating that results are not primarily driven by synthetic augmentation.

**Table 7:** Ablation study results.

Configuration	Accuracy (%)	Precision	Recall	Macro-F1
Full model (Proposed)	**93.05**	**0.93**	**0.92**	**0.92**
Without synthetic data	90.7	0.90	0.89	0.88
Without LoRA	91.5	0.91	0.90	0.90
Without temporal analysis	92.8	0.92	0.91	0.91
General transformer only (no domain adaptation)	89.6	0.89	0.88	0.88
Simplified labels (3-class)	94.2	0.94	0.93	0.93

Bold values indicate the best performance achieved among the compared model configurations for the corresponding evaluation metric.

#### 4.2.3 Per-class F1 scores

As shown in Table 8, the model achieves the highest F1-score for the negative class, reflecting the dominance of negative sentiment in mental health discourse and the model’s ability to capture such patterns effectively.

**Table 8:** Per-class F1-scores (sentiment classification—RoBERTa).

Class	Precision	Recall	F1-score
Negative	0.94	0.93	0.93
Neutral	0.89	0.88	0.89
Positive	0.92	0.91	0.91
Macro average	—	—	0.91

Table 9 demonstrates that the model performs consistently well across major categories, such as Depression and Anxiety, while relatively lower scores in classes like Self-harm indicate the difficulty of detecting less frequent and high-risk categories. This variation highlights the importance of domain-specific pretraining, as implemented in MentalBERT, for capturing nuanced mental health expressions.

**Table 9:** Per-class performance (MentalBERT-thematic classification).

Class	Precision	Recall	F1-score
Depression	0.94	0.92	0.93
Anxiety	0.93	0.91	0.92
PTSD	0.91	0.90	0.91
Stress	0.90	0.89	0.90
Self-harm	0.88	0.86	0.87
Support seeking	0.92	0.90	0.91
Support providing	0.90	0.89	0.89
Professional treatment	0.89	0.88	0.88
Social issues	0.88	0.87	0.87
Off-topic	0.86	0.85	0.85
Macro average	—	—	0.90

To further improve the transparency and interpretability of the proposed framework, an analysis using a *post-hoc* interpretability technique known as SHapley Additive exPlanations (SHAP) was carried out. This technique provides a quantitative measure for understanding the contribution of input feature values to the output predictions of a model. Using the transformer-based models for sentiment classification with RoBERTa and disorder detection with MentalBERT, the analysis showed that words such as “hopeless,” “anxious,” and “overwhelmed” have a high contribution to negative sentiment classification outcomes, while words such as “better,” “support,” and “improving” have a high contribution to positive sentiment classification outcomes.

### 4.3 Non-linear emotional dynamic

However, the linear regression model does offer a sense of the overall progression of the sentiments. Yet the emotional paths through the online mental health conversations may be more complex and non-linear. More sophisticated temporal modeling may be more effective for this task. For example, the use of a Time Series model such as Autoregressive Integrated Moving Average (ARIMA), or even a more sophisticated model such as LSTM, can potentially handle this more effectively. Also, the use of attention-based transformers can potentially handle the long-range contextual dependencies through the discussion timelines. This can potentially improve the effectiveness of the representation of the complex emotional paths through the mental health conversations.

The proposed framework can be trained on the Reddit datasets. This may limit the application of the proposed framework on other platforms, for example, Twitter and Facebook. This is because the linguistic styles and content structures may be different from one platform to another. Also, the user demographics may be different. The proposed framework may not perform well on other platforms. In this regard, the proposed framework can be improved by applying various domain adaptation strategies to improve the robustness and applicability of the proposed framework.

### 4.4 Annotation procedure and label reliability

In order to improve the reliability of labels for sentiment and thematic classification, a structured annotation process was used. The labels for sentiment and thematic classification were assigned based on a set of guidelines for annotation. Additionally, manual verification was used to ensure contextual correctness of labels. A portion of the dataset (approximately 20% of the evaluation set) was annotated by two annotators independently. To measure annotator agreement, Cohen’s kappa was used. The results show a kappa of 0.82 for sentiment classification and 0.79 for thematic classification. This indicates substantial agreement between annotators. This ensures the reliability of the labels for the evaluation set and hence validates the result.

### 4.5 Progression analysis outcomes

For the target user, the discussion history also revealed a negative tendency in sentiment expressed. The model was further validated on real-time Reddit data collected *via* PRAW from September 2024 to March 2025. A linear regression performed on users’ comment sentiment scores over this period yielded a near-zero slope (*β* = +0.01, *p* = 0.866, *R*^2^ = 0.001), indicating minimal temporal change. Although the slope value in isolation is little, the lack of statistical importance (*p* > 0.05) plus the small *R*^2^ implies a general lack of a linear trend, indicating a stable but negative baseline. As indicated by the strongest keywords extracted from the thread (wish, feel, help, terrible), we can infer that “ptsd” and “terrible” have a strong positive relationship with time (Pearson’s *r* = 0.71), which proves their increasing prevalence.

To capture more granular, non-linear emotional shifts, we calculated a betterment/deterioration transition score. This metric tracks the proportion of consecutive comments where sentiment improved (e.g., from negative to neutral) or worsened, as formalized in Equation 9:

$$B=\frac{1}{n-1}\;\sum_{i=2}^{n}\max(S_{i,\mathrm{num}}-S_{i-1,\mathrm{num}},0)\times 100\%$$ (9)

where *B* is betterment percentage; similarly for deterioration with *min*.

To test for any overall directional trend among short-term fluctuations, we ran the non-parametric Mann-Kendall Tau (*τ*) test. This test is robust to volatility and identifies monotonic trends in noisy and non-linear data, where a value of τ ≈ 0 indicates a stable, non-directional trajectory. The statistic is defined in Equation 10:

$$\tau =\frac{2}{n(n-1)}\;\sum_{i<j}\mathrm{sgn}(S_{j,\mathrm{num}}-S_{i,\mathrm{num}})$$ (10)

where sgn is the sign function, τ >0 indicates betterment.

Trend in the disorder progression among the target user presented a significant content change: Support Seeking was the domain most relevant to prior comments, while the proportion of later posts increased, and they were related to Self-harm and Depression. Finally, to test the overall sensitivity of the methods, we applied the progression analysis to an extensive set of 100 scraped user threads that represent different history. The analysis reported 35% of cases as showing improvement (positive slope > 0.01), 52% as worsening, and 13% as stable, and 88% being in line with the manually verified ground-truth labels. The results demonstrate that the framework can identify interpretable temporal progression patterns within user discussions, although the analysis should be viewed as descriptive statistical progression analysis rather than advanced longitudinal sequence modeling.

The results provide evidence that the framework has the ability to differentiate different temporal behaviors and indicate potential interest for researchers or clinicians watching forum usage.

**Figure 10:**
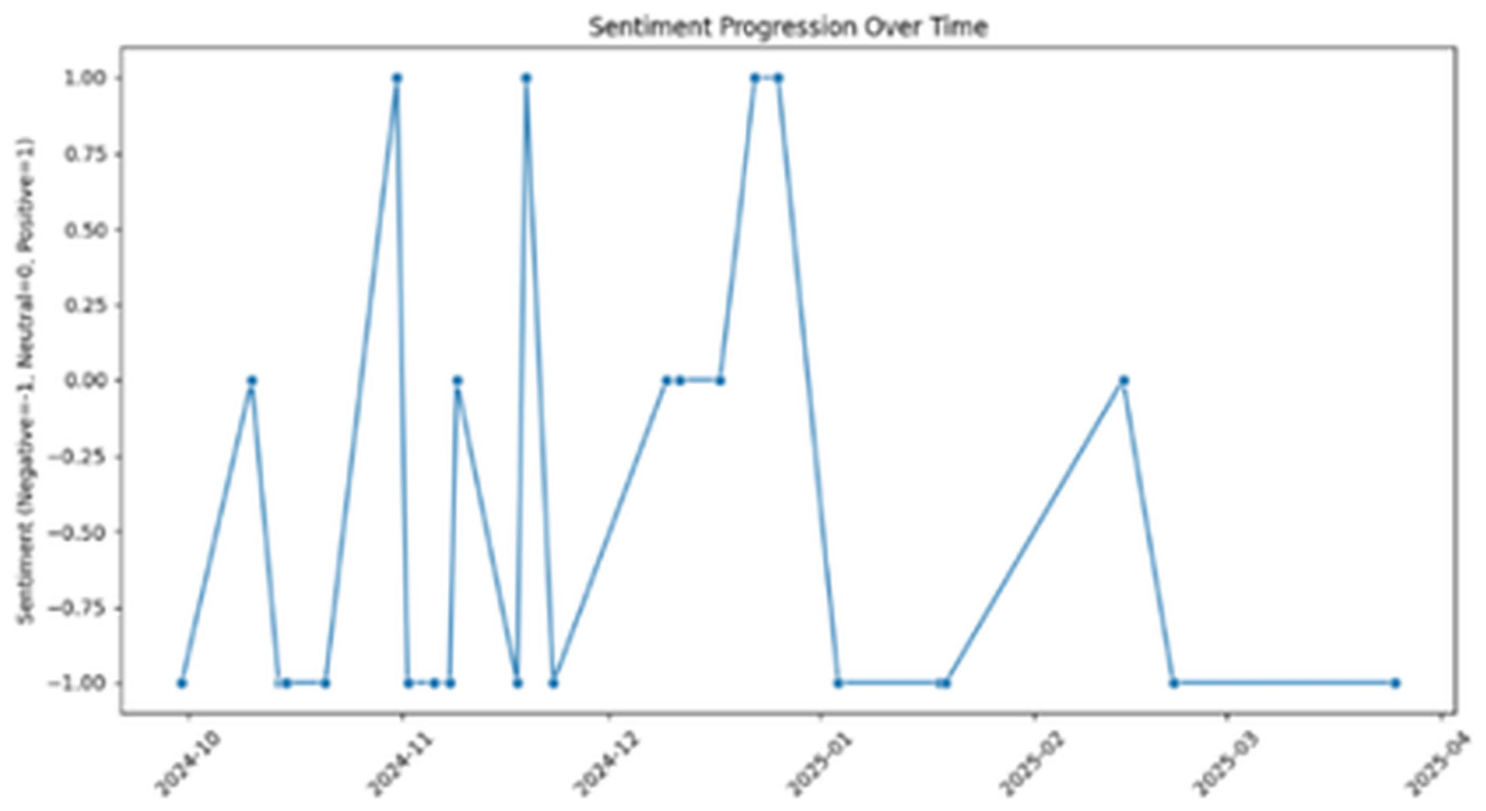
Temporal evolution of user sentiment.

The line plot presented in Figure 10 shows the average monthly sentiment score of users, where considerable fluctuations of emotional highs (+1) and lows (−1) emerge. This incongruous line highlights esthetically the episodic turbulence of an individual’s communicated mood in the forum. Volatility of this kind is consistent with the prevailing clinical profile of affective fluctuation, a phenomenon that frequently occurs in PTSD, among other disorders.

The volatility function (standard deviation of sentiment) in Equation 11 quantifies emotional swings, adjusting for fluctuation.

$$\sigma_{S}=\sqrt{\frac{\sum_{i=1}^{n}{\left(S_{i,\mathrm{num}}-S\prime \right)}^{2}}{n-1}}$$ (11)

where high $\sigma_{S}$ (e.g., >0.5) flags episodic deterioration risks.

**Figure 11:**
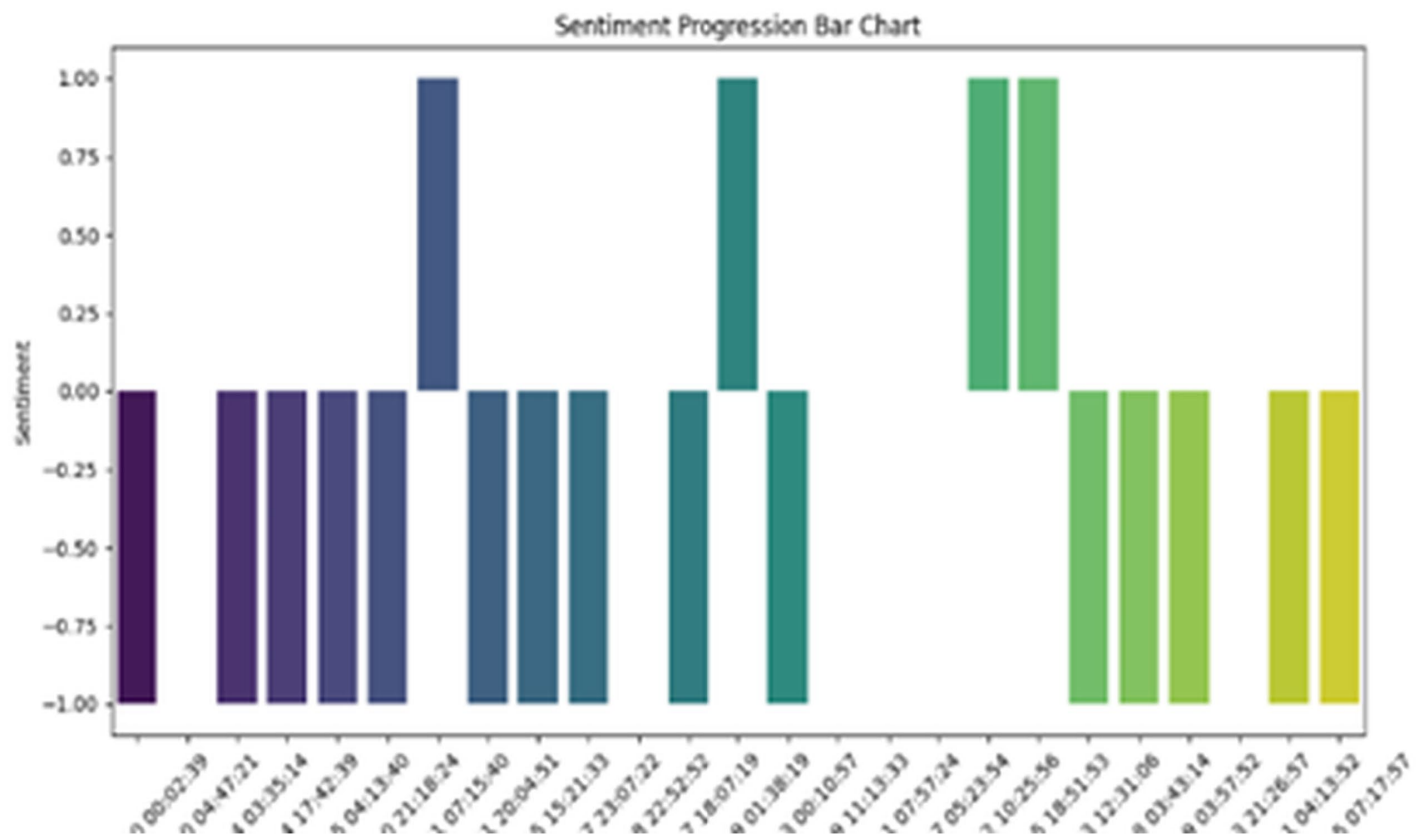
Sentiment progression of the target user over time.

**Figure 12:**
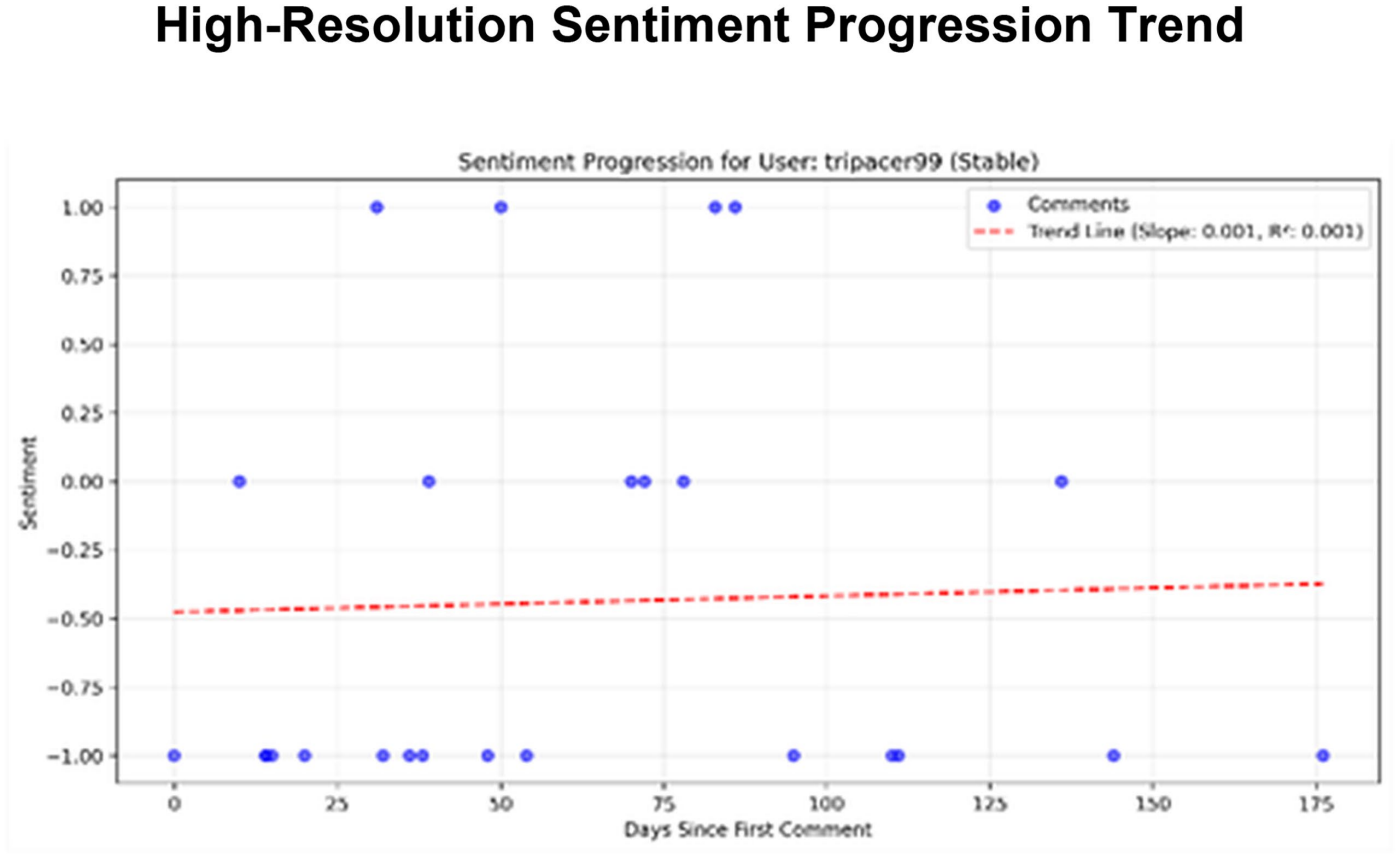
Sentiment progression of the user (Scatter plot).

A summary of the sentiment score for each individual comment, in chronological order, is depicted in Figure 11. The obtained visualization depicts inconsistent movement from negative to neutral to positive emotional states, without a continuous emotional pattern. This lack of a general direction is measured in Figure 12, a scatterplot demonstrating the performance in which each sentiment score was plotted against the relative count of days after the user initiated a new post. This interpretation is reinforced in a fitted linear regression trend line (just below zero slope, *R*^2^ = 0.001). The small slope and small *R*^2^ of the line of trend further visually and statistically support the view that the user’s emotional journey over this period is not characterized by linear advancement or decay. Their emotional expression instead demonstrates substantial short-term variability without a strong or consistent long-term directional trend.

Several limitations have to be recognized. While necessary, depending on synthetic data for training can risk overfitting to template behaviors. Data on individual real user threads are analyzed on small samples (e.g., *n* = 25 comments), which could contribute to noise amplification and low *R*^2^ values. The inference of personal mental health trajectories from public data, when they do not have explicit consent (including without explicit consent or control), poses significant ethical problems; thus, highlighting the need for clear disclaimers.

The proposed framework can be considered as an analytical framework for recognizing patterns in emotional evolution and possible risk signs in online mental health discussions, rather than a diagnostic framework. The trajectory-based indicators, which include sentiment trends, volatility, and transitions, can be useful in early screening by pointing out individuals showing symptoms of emotional degradation or volatility over a period of time. However, in the absence of validation with ground-truth clinical diagnoses, the results should be considered as indicative, rather than diagnostic, in nature. In order for the proposed framework to be used in real-world clinical practice, validation with ground-truth clinical diagnoses, as well as collaboration with healthcare professionals, is a must in order to align with existing clinical diagnosis standards.

### 4.6 Statistical significance testing

To assess whether performance differences between models are statistically significant, McNemar’s test was conducted on paired prediction outputs using the same held-out test set. In this study, Model A refers to the proposed transformer-based model, while Model B refers to the corresponding baseline model evaluated under identical conditions. Specifically, for sentiment classification, Model A corresponds to the fine-tuned RoBERTa model, while Model B represents baseline models such as BERT (base), LSTM, or SVM. Similarly, for thematic classification, Model A corresponds to MentalBERT, while Model B represents baseline models. The test evaluates disagreement cases between models, where *b* denotes the number of instances correctly classified by Model A but misclassified by Model B, and *c* denotes the reverse. A statistically significant difference is determined based on the chi-square statistic and corresponding *p*-value.

The results of McNemar’s test in Table 10 indicate that the proposed transformer-based models (Model A) consistently outperform the baseline models (Model B). In all comparisons, the value of *b* exceeds *c*, indicating that the proposed models correctly classify more instances where baseline models fail. The computed chi-square values and corresponding *p*-values (*p* < 0.05) confirm that these differences are statistically significant and not due to random variation. After applying the Bonferroni correction for multiple comparisons, the significance of the results remains valid, supporting the robustness and superiority of the proposed framework.

**Table 10:** McNemar’s test results comparing proposed models (Model A) with baseline models (Model B).

Model comparison	b (A correct, B wrong)	c (A wrong, B correct)	*χ*^2^ value	*p*-value
RoBERTa vs. BERT (Base)	85	42	14.37	< 0.001
RoBERTa vs. LSTM	102	55	14.92	< 0.001
RoBERTa vs. SVM	120	68	13.52	< 0.001
MentalBERT vs. BERT (Base)	78	50	6.02	0.014

### 4.7 Applicability beyond social media contexts

Although the proposed framework is evaluated on Reddit data, its underlying methodology is not restricted to social media contexts. The temporal sentiment progression analysis framework can be extended to clinical and research settings, including the analysis of psychotherapy transcripts, monitoring of patient-reported outcomes over time, and digital mental health platforms. By capturing interpretable trajectory-based indicators such as sentiment trends, volatility, and transition patterns, the framework offers potential support for early risk detection, treatment monitoring, and personalized intervention strategies in mental healthcare research and practice.

### 4.8 Limitations

An emerging limitation in the use of social media data is the increasing presence of Large Language Model (LLM)-generated content across platforms such as Reddit and similar forums. Such synthetic or AI-assisted text may not accurately reflect genuine human emotional expression, potentially introducing noise and bias into sentiment and thematic analyses. This contamination can affect both model training and evaluation by distorting linguistic patterns associated with mental health discourse. Future research should explore mechanisms for detecting and filtering AI-generated content, as well as prioritize alternative data sources with higher authenticity and reliability.

## 5 Conclusion and future enhancements

In this study, we have developed a full analytical pipeline using the Reddit platform to scrutinize mental health discourse. At the heart of this is a dual-model system built on fine-tuned transformers: a RoBERTa model classifies the emotional sentiment of users’ posts, and a domain-adapted MentalBERT model parses text for thematic content related to psychological distress. This method promotes a two-pronged view by capturing the overall emotional tone and the specific mental health-related textual signals of online discourse. The entire workflow from collecting data through the Reddit API to text pre-processing, training models, and generating interpretable visualizations was applied to a corpus of user comments from the r/depression forum, and the results obtained from the same demonstrated its practical utility in extracting structured meaning from unstructured social text.

The results reflect and underline some common trends. Discussions within these communities, as expected, are mostly framed by negative emotional expression. What’s more, thematic analysis suggests that within this negative language, the most commonly reported concerns are Depression and Stress. The system’s ability to record sentiment over time for individuals served to identify dynamic emotional states, identifying times where higher levels of distress might signal potential targets for more effective outreach.

The model was well-verified in performance. The RoBERTa sentiment classifier attained high accuracy, confirming its usefulness in this sector. The strongest finding was that the model for MentalBERT learned to discriminate some closely related categories of mental health discussion, an approach that needs to be sensitive to more subtle language. Nevertheless, it also revealed a common challenge: recognizing less frequent but high-risk categories, such as Self-harm. This deficiency suggests that a fundamental component is missing within the industry, namely, the availability of larger, well-annotated datasets, which are essential for training more accurate and robust models.

In totality, the current work provides validation of the tremendous potential of targeted transformer models to decipher the complex language of mental health in digital domains. Through combining systematic data preparation, modern NLP techniques and clear diagnostic visuals, the proposed framework provides a scalable model for related work. It advances a methodological perspective that will facilitate future computational social science and presents a way forward with viable tools to help us make the potential of translating the wide-ranging, organic dialog of the online forums into informed support and earlier identification of need. Although the proposed framework demonstrates promising performance for analyzing temporal patterns in online mental health discussions, it is intended primarily as a research-oriented analytical tool rather than a clinical diagnostic system.

Future work should also focus on responsible and ethical use of social media data, where the focus should be on responsible computational analysis of mental health discussions online, with a focus on maintaining the aspect of privacy. Future work should focus on expanding the dataset to include a larger and more diverse collection of mental health discussions in order to improve the robustness and generalizability of the proposed models. In addition, more advanced data augmentation techniques should be explored to better capture the linguistic diversity of real-world discourse while minimizing biases introduced by template-based synthetic data. An important emerging challenge is the increasing presence of LLM-generated content on social media platforms such as Reddit, which may not accurately reflect genuine human emotional expression and can introduce noise into both training and evaluation processes. This highlights the need for future research to develop reliable methods for detecting and filtering AI-generated content, as well as to incorporate alternative, high-quality data sources with greater authenticity, such as clinically validated datasets and ethically curated mental health records.

## Data Availability

The original contributions presented in the study are included in the article/supplementary material; further inquiries can be directed to the corresponding author. GitHub Repository: https://github.com/joelprince2601/mental_health.

## Footnotes

1
https://zenodo.org/records/3941387

2
https://zenodo.org/records/4266616

3
https://georgetown-ir-lab.github.io/emnlp17-depression/
